# DUX4-induced dsRNA and MYC mRNA stabilization activate apoptotic pathways in human cell models of facioscapulohumeral dystrophy

**DOI:** 10.1371/journal.pgen.1006658

**Published:** 2017-03-08

**Authors:** Sean C. Shadle, Jun Wen Zhong, Amy E. Campbell, Melissa L. Conerly, Sujatha Jagannathan, Chao-Jen Wong, Timothy D. Morello, Silvère M. van der Maarel, Stephen J. Tapscott

**Affiliations:** 1 Molecular and Cellular Biology Program, University of Washington, Seattle, Washington, United States of America; 2 Division of Human Biology, Fred Hutchinson Cancer Research Center, Seattle, Washington, United States of America; 3 Division of Basic Sciences, Fred Hutchinson Cancer Research Center, Seattle, Washington, United States of America; 4 Computational Biology Program, Public Health Sciences Division, Fred Hutchinson Cancer Research Center, Seattle, Washington, United States of America; 5 Department of Human Genetics, Leiden University Medical Center, Leiden, Netherlands; The Jackson Laboratory, UNITED STATES

## Abstract

Facioscapulohumeral dystrophy (FSHD) is caused by the mis-expression of DUX4 in skeletal muscle cells. DUX4 is a transcription factor that activates genes normally associated with stem cell biology and its mis-expression in FSHD cells results in apoptosis. To identify genes and pathways necessary for DUX4-mediated apoptosis, we performed an siRNA screen in an RD rhabdomyosarcoma cell line with an inducible *DUX4* transgene. Our screen identified components of the MYC-mediated apoptotic pathway and the double-stranded RNA (dsRNA) innate immune response pathway as mediators of DUX4-induced apoptosis. Further investigation revealed that DUX4 expression led to increased MYC mRNA, accumulation of nuclear dsRNA foci, and activation of the dsRNA response pathway in both RD cells and human myoblasts. Nuclear dsRNA foci were associated with aggregation of the exon junction complex component EIF4A3. The elevation of MYC mRNA, dsRNA accumulation, and EIF4A3 nuclear aggregates in FSHD muscle cells suggest that these processes might contribute to FSHD pathophysiology.

## Author summary

Facioscapulohumeral dystrophy (FSHD) is a common form of muscular dystrophy which is currently untreatable. It is caused by the inappropriate expression in skeletal muscle of the gene *DUX4* that encodes a transcription factor normally expressed in some stem cells. When DUX4 is expressed in cultured human or mouse skeletal muscle cells, it activates a program of cell death. Knowing the molecular basis for the cell death induced by DUX4 is important to determine the mechanism of muscle damage in FSHD. We used a molecular screening approach to identify genes and pathways necessary for DUX4 to induce the cell death program. We found that DUX4 activated a known MYC-induced cell death pathway, at least in part through stabilization of MYC mRNA. We also found that DUX4 expression led to an accumulation of double stranded RNAs (dsRNAs) that induced a cell death pathway evolved to protect against viral infections. This dsRNA accumulation was accompanied by aggregation of the EIF4A3 protein, a factor involved in mRNA surveillance and decay, which may provide a partial mechanism for how DUX4 can inhibit RNA quality control pathways in cells. Because FSHD muscle cells have increased MYC mRNA, dsRNA accumulation, and EIF4A3 nuclear aggregates, we conclude that these processes might contribute to FSHD pathophysiology.

## Introduction

Facioscapulohumeral dystrophy (FSHD) is a progressive muscular dystrophy caused by mis-expression of the double-homeobox transcription factor DUX4 in skeletal muscle [1]. Normally, DUX4 is not expressed in skeletal muscle nor in most somatic tissues examined [2,3]. Ectopic expression of DUX4 in human and mouse cell lines as well as *in vivo* injection of DUX4 adenovirus into mouse muscle leads to rapid cellular apoptosis [4,5]. This cell death is dependent on the transcriptional activity of DUX4 because expression of DUX4 with mutations in the DNA binding domain or trans-activation domain do not exhibit toxicity [5,6]. More recently, it was demonstrated that endogenous levels of DUX4 produced in FSHD muscle cells similarly causes cellular death [7].

Apoptosis is known to be a critical cellular process for both tissue homeostasis as well as vertebrate ontogeny where cells in developing organs follow the general guidelines of “proliferation, differentiation and demolition” [8]. It is also appreciated that cellular apoptosis, outside of normal homeostatic or developmental contexts, is involved in autoimmune and neurological diseases [9,10]. As evidenced by numerous mouse knockout lines, programmed cell death is required for productive sperm development where excess or abnormal germ cells are constantly culled to ensure adequate space and nutrients [11]. Previously, immunodetection has identified DUX4 expression in cells in the seminiferous tubule, morphologically resembling spermatogonia or primary spermatocytes [2] and in the thymus [3], both tissues with high rates of apoptosis. Thus, it is possible that expression of DUX4 in skeletal muscle inappropriately activates a program of apoptosis that might otherwise be a 'normal' consequence of DUX4 expression during developmental processes.

As a transcription factor, DUX4 activates many genes that are expressed in stem cells and in the germline [12]. The long terminal repeat (LTR) of a subset of human endogenous retroviruses (ERVs) contain the DUX4 binding motif, and DUX4 binds and activates their transcription, occasionally creating a novel transcription start site for adjacent genes [13]. DUX4 expression also represses the innate immune response [12] and the nonsense mediated decay (NMD) pathway [14], leading to an accumulation of normally degraded RNAs. However, it is not currently understood whether the changes in RNA stabilization following DUX4 expression lead to apoptosis. It has been demonstrated that FSHD muscle biopsies exhibit oxidative stress and mitochondrial dysfunction compared to control biopsies [15] and small molecule screens have demonstrated that compounds which protect cells from oxidative damage also protect against DUX4 toxicity [6,16]. It was also previously shown that *Tp53* knockout mice were protected from the effects of DUX4 delivered to skeletal muscle by AAV transduction and that a P53 inhibitor decreased DUX4 toxicity in human HEK293 cells [5]. However, the DUX4-induced apoptotic pathways relevant to human skeletal muscle and FSHD remain poorly understood.

We conducted a small interfering RNA (siRNA) screen to identify genes and pathways necessary for DUX4 toxicity. This screen identified the MYC-mediated apoptotic pathway and components of the double-stranded RNA (dsRNA) innate immune response as necessary for DUX4-induced apoptosis. We found that DUX4 expression resulted in the stabilization of several mRNAs, including MYC, and the accumulation of nuclear dsRNAs. Stabilization of MYC mRNA was associated with a dramatic increase in MYC protein levels and the activation of genes in the MYC-mediated apoptosis pathway, including *BCL2L11* and *EGR1*; whereas the accumulation of dsRNA was associated with nuclear aggregation of EIF4A3 and phosphorylation of the kinase EIF2AK2/PKR and its downstream target, the eukaryotic translational initiation factor subunit EIF2S1/eIF-2α. The similar elevation of MYC mRNA, dsRNA accumulation, and EIF4A3 nuclear foci in FSHD muscle cells suggest that these processes might contribute to FSHD pathophysiology.

## Results

### siRNA screen identifies candidate genes necessary for DUX4-induced cell death

Although a prior study had identified *TP53* as necessary for DUX4-induced cell death in some cells [5], we found that human myoblasts with CRISPR mutated *TP53* and the RD rhabdomyosarcoma cell line that does not contain a functional *TP53* allele [17,18] both succumbed to DUX4-induced cell death as efficiently as primary human myoblasts (S1A–S1D Fig). To establish a screen for genes necessary for DUX4-induced apoptosis, we transduced the RD cell line with a lentiviral vector encoding a doxycycline-inducible DUX4 coding sequence and puromycin selectable marker (see S2A Fig for schematic) and generated a clonal rhabdomyosarcoma cell line (RD-DUX4i) with robust doxycycline-inducible expression of DUX4 that showed cellular toxicity at 24 hours after DUX4 induction (S2B Fig) and more than 95% cell death by 48 hours, as determined by the ATP-based CellTiter-Glo assay (S2C Fig). The dying cells exhibited an increase in activated caspase 3/7 (S1A Fig) indicating that DUX4 induction leads to apoptosis in the P53 deficient RD cells.

To identify genes and pathways necessary for DUX4-induced toxicity in RD cells we measured cell survival following transfection of an siRNA library targeting 6,961 genes in the human “druggable” genome with a pool of four siRNAs per target gene. The parameters for the screen are summarized in S2D Fig and were optimized as in S3A Fig. As positive controls, we used two unique siRNAs targeting DUX4 which had different efficacies of knockdown (S3B Fig) and, consequently, different effects on cell viability after DUX4 induction (S2E Fig). Transfections were performed in triplicate and cell survival following DUX4 induction was measured by CellTiter-Glo.

Using a stringent mean Z-score threshold of 3.0, 69 siRNA pools significantly increased cell viability in response to DUX4. Targets that exceeded this threshold included some genes previously implicated in cellular apoptosis, for example: Death effector domain containing 2 (*DEDD2*), Cell death-inducing DFFA-like effector a (*CIDEA*), MutS homolog 2 (*MSH2*), C-MYC (*MYC*) and its dimerization partner *MAX*, and *RNASEL*, a mediator of cellular antiviral defense. Using a more traditional Z-score cutoff of 2.0, 353 siRNA pools enhanced cell viability after DUX4 induction. In addition, 30 siRNAs had a Z-score of less than -2.0, suggesting that these genes might protect cells from DUX4-induced death. Among these targets were the DUX4 target genes, *TRIM51* (also known as *SPRYD5*) and *TRIM43* as well as the anti-apoptotic *BCL2L1* (also known as *BCL-X*) that can suppress MYC-mediated cell death [19]. As expected, siRNAs targeting *TP53* had no effect on cell viabilty (Z-score = -0.03). Network analysis placed MYC/MAX as a 'hub' of protein-protein interactions among the candidate genes with an absolute value Z-score cutoff of 2.0 (S3C Fig). The general results of the screen are summarized in S2E Fig, and the full ranked list is available in S1 Table.

**Fig 1:**
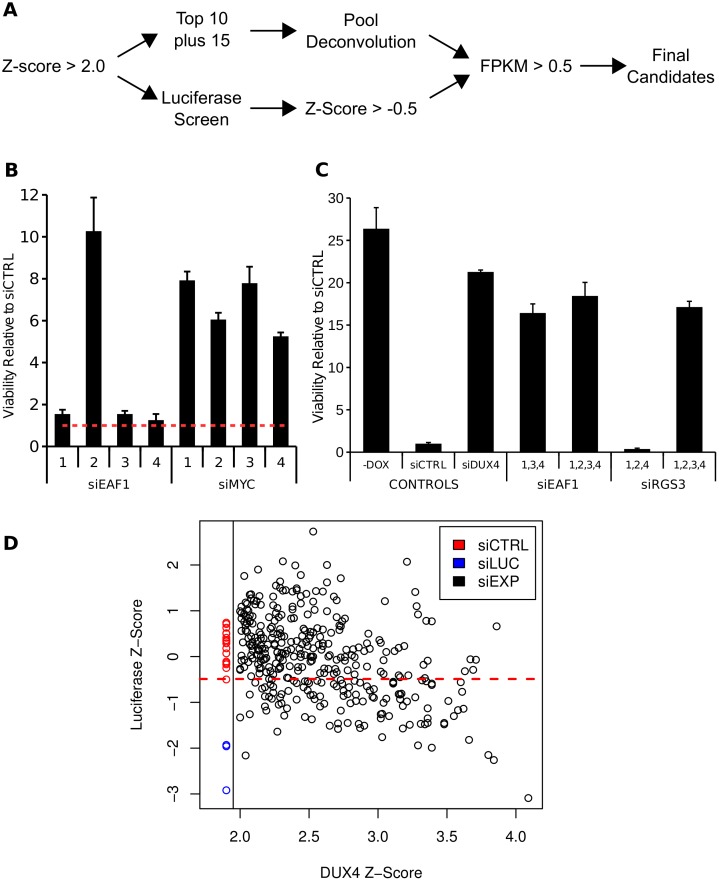
Elimination of siRNA pools with possible off-target activities. (A) Schematic of secondary screening used to filter out unwanted targets from the primary siRNA screen. (B) Example of how siRNA pools from the primary screen were deconvoluted to determine whether the effect on cell viability was dominated by a single siRNA which would suggest off-targeting. In this example, EAF1 has a single dominant siRNA that rescues, whereas multiple siRNAs to MYC rescue. These data depict cell viability using the CellTiter-Glo assay. (C) Pooling of poorly performing siRNAs can 'synergize' to rescue from DUX4 toxicity as with siEAF1 whereas others are clearly dominated by a single siRNA as in siRGS3. These data depict cell viability using the CellTiter-Glo assay. (D) Scatter-plot of mean Z-scores from the original DUX4 siRNA screen against mean Z-scores produced in the Luciferase screen to determine the siRNA pools that inhibited doxycycline induction of the transgene. Error bars for (B) and (C) depict standard deviation of the mean of three replicate wells.

We proceeded to validate candidate siRNA pools identified in the initial screen using a strategy outlined in Fig 1A. As a first validation step we transfected RD-DUX4i cells with the ten siRNA pools with the highest Z-scores plus 15 additional pools with Z-scores ranging from 1.93 to 3.61. All 25 pools increased viability over the non-silencing control siRNA, indicating that pools identified in the primary screen reproducibly enhanced cell survival after induction of DUX4 (S4A Fig).

As a second validation step we de-convoluted these 25 pools of four individual siRNAs to determine whether multiple siRNAs within each pool contributed to the increased survival of RD cells rather than a single siRNA which might indicate a nonspecific, off-target effect. Of the 25 pools retested, 21 had more than one siRNA that enhanced cell survival by 2-fold or greater (Fig 1B and S4B Fig). For the four pools with a single siRNA that rescued viability, we tested whether the individual non-rescuing siRNAs from that pool might rescue if pooled together, reasoning that synergy among the individually non-rescuing siRNAs might indicate on-target activity. Re-transfection of these non-rescuing siRNAs as pools of three revealed that more than one siRNA against two genes (*EAF1* and *TLR5*) rescued from DUX4 toxicity (Fig 1C and S4C Fig), whereas for the other two genes (*TLL1* and *RGS3*), only a single siRNA had rescuing activity.

Further analysis showed that the rescue conferred by siRNAs in pools where there was a single rescuing siRNA, such as siRGS3, was correlated with the inhibition of doxycycline induction of DUX4 and of a doxycyline inducible luciferase transgene in RD cells (RD-LUCi cells; S4D Fig). This indicated that some of the siRNA pools in the original screen likely achieved RD-DUX4i cell survival and a high Z-score because a single siRNA in the pool inhibited the doxycycline induction of DUX4 through unknown mechanisms.

Therefore, as a third validation step we tested for the inhibition of the doxycycline induction pathway on the entire set of siRNA pools with an original Z-score ≥ 2.0 using the RD-LUCi cells. 110 of our initial 353 pools suppressed luciferase induction based on a cutoff Z-score of ≤ -0.5 (Fig 1D and S2 Table), the minimum score of the 16 scrambled control siRNAs used in this secondary screen. For example, the RGS3 siRNA pool, which we had already determined had a single siRNA that suppressed doxycycline induction of luciferase, had a mean Z-score of -3.09. Overall, there was a modest but significant inverse correlation between the RD-DUX4i screen Z-scores and the RD-LUCi screen Z-scores (Spearman's rho = -0.396), indicating that some of the pools identified in the original screen were likely secondary to inhibiting the doxycycline induction pathway. Therefore, we eliminated the 110 genes targeted by pools that depress luciferase induction by more than the non-silencing control.

As a final filter, we performed RNA-sequencing on RD-DUX4i cells and required that the targeted mRNA must be expressed at greater than an average FPKM (fragments per kilobase of transcript per million mapped reads) of 0.5 to filter out low or non-expressed genes. For example, although TLR5 apparently rescued based on initial validation criteria (see above), it was expressed below the threshold level and was therefore eliminated. Of the original set of 6,961 genes targeted by the library, 16 genes with a Z-score ≥ 3.0 passed our validation and filtering steps (Table 1). The final list of filtered targets with Z-score ≥ 2.0 is summarized in S3 Table.

**Table 1:** Top 16 filtered targets with Z-score > 3.0 from RD-DUX4i siRNA screen.

Target ID	DUX4i Z-score	LUCi Z-score	AVG FPKM^a^-DOX	AVG FPKM^a^ +DOX	Validation % of -DOX^b^	Mean normalized Fold Rescue^c^
FOSB	3.86	0.66	0.16	8.59	54.7	75.17
RNASEL	3.71	-0.07	0.94	0.54	52.4	63.50
MYC	3.69	-0.29	29.87	696.89	63.9	62.60
FXN	3.67	-0.06	3.51	0.77	61.6	61.70
EAF1	3.63	-0.32	5.89	19.27	34.0	58.60
PHF20	3.39	0.77	4.93	1.36	38.3	44.93
NLGN2	3.36	-0.09	8.85	1.77	NA	43.17
TGS1	3.35	0.78	10.11	12.56	NA	43.10
HSD17B4	3.29	0.92	5.57	2.09	NA	39.97
ITPK1	3.28	1.1	1.41	0.44	NA	39.60
SGK196	3.19	0.23	4.64	2.52	NA	36.47
CDC20	3.15	-0.41	79.51	358.73	NA	33.87
QSOX1	3.15	0.19	7.54	4.37	NA	33.87
THOP1	3.06	0.1	11.19	3.34	NA	30.87
FBN2	3.04	-0.08	0.91	0.51	NA	30.37
APH1A	3.02	-0.33	15.96	4.79	NA	30.00

^a^AVG FPKM, mean fragments per kilobase of transcript per million mapped reads across three replicates.

^b^Validation % of -DOX, mean CellTiter-Glo determined viability of the indicated target as a percentage of the no doxycycline control in the validation experiment as in S3A Fig. 'NA' indicates that the given target was not retested. For comparison purposes, the scrambled control siRNA viability was 4.7% of the -DOX control.

^c^Mean Normalized Fold Rescue, the average ratio between raw target fluorescent measurements from the original screen and the sample median for the 384-well plate as a whole. For comparison purposes, the average ratio for the more robust DUX4 siRNA was 80.0.

### DUX4 inhibits MYC mRNA degradation and activates a MYC-mediated apoptotic pathway

The filtered targets meeting a Z-score threshold of 3.0 included several genes that broadly regulate RNA transcription (*MYC*, *FOSB*, *EAF1*, *PHF20*, *CDC20*), protein translation (*TGS1*) [20], or mitochondrial function (*FXN*) [21]. MAX, the obligate heterodimer of MYC, also appeared as a candidate target in the initial screen, with a Z-score exceeding 3.0, although MAX was subsequently eliminated from the final candidate list because the pool of siRNAs to MAX demonstrated a modest reduction in doxycycline induction of the luciferase transgene (Z-score = -0.93). It is interesting to note that many of the aforementioned genes were upregulated in our RNA-seq data in RD cells overexpressing DUX4: *FOSB* (~92 fold), *MYC* (~56 fold), *EAF1* (~8 fold) and *CDC20* (~11 fold). Although siRNAs against MYC had a modest effect on transgene expression (luciferase Z-score = -0.29), the siRNAs also rescued viability following lentiviral transduction of a constitutively expressed DUX4 in cells with DUX4 protein levels and nuclear localization equivalent to the controls (S4E–S4G Fig). Therefore, we decided to investigate the MYC-mediated apoptosis pathway because DUX4 expression resulted in a dramatically increased expression of *MYC* as well as a set of genes that facilitate a program of enhanced cellular growth or metabolism.

**Fig 2:**
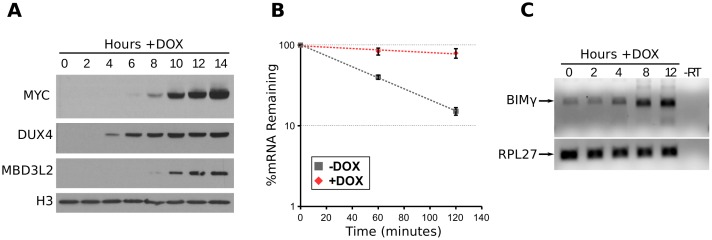
DUX4 increases MYC protein through MYC mRNA stabilization and induces the BIMγ isoform of BCL2L11. (A) Western blot of RD-DUX4i cell extracts following DUX4 induction at the indicated timepoints and using the indicated antibodies. (B) Semi-log plot of MYC mRNA levels in RD-DUX4i cells pretreated +/- doxycyline for 8 hours and in the presence of DRB for the indicated time points. Data are normalized to 18s rRNA levels which we reasoned would not be transcriptionally repressed by DRB, an RNA polymerase II inhibitor. Error bars depict standard deviation of the mean of three independent experiments. (C) RT-PCR of the BIMγ isoform following DUX4 expression in RD-DUX4i cells with the constitutively expressed RPL27 as a control.

We first verified that MYC protein was upregulated following DUX4 expression. Western blotting and RT-qPCR confirmed that MYC protein and RNA levels increased dramatically following DUX4 expression (Fig 2A, S5A and S5B Fig), tracking closely with a direct target of the DUX4 transcription factor, MBD3L2 (Fig 2A). The increase in MYC did not appear to correspond to a large change in MYC protein stability as determined by western blotting after cycloheximide (CHX) treatment to block de novo translation (S5C Fig). There was no clear evidence of direct transcriptional activation of MYC by DUX4 based on prior chromatin immunoprecipitation sequencing (ChIP-seq) data [13] which did not identify a DUX4 binding site near the *MYC* gene (S5A Fig), although PolII ChIP showed a modest increase in promoter-associated and elongating PolII occupancy at the *MYC* promoter (S5D Fig), about 2-fold and 4-fold, respectively. Most dramatic, however, was a clear enhancement of MYC mRNA stability following DUX4 induction as evidenced by the increased half-life from roughly 44 minutes to 360 minutes in DUX4-expressing cells (Fig 2B). To determine whether this increase in mRNA stability was specific to MYC or a more general effect on mRNA stability, we assessed the stability of two other labile mRNAs, JUN and CITED2, following DUX4 expression. Although these results were less dramatic, the trend indicated an increase in mRNA stability of both genes following DUX4 expression (CITED2 from approximately 45 to 182 minutes and JUN from 52 to 140 minutes; S5E Fig). We conclude that, although the effect might not be specific to MYC, a major contribution to the increased MYC levels was likely through inhibition of mRNA degradation. These results are in agreement with a more general inhibition of mRNA degradation pathways in DUX4-expressing cells, similar to the previously demonstrated inhibition of NMD which leads to the stabilization of numerous labile mRNAs [14].

Studies in other cells have shown that the MYC-mediated pathway of apoptosis involves MYC activation of EGR1, and then MYC and EGR1 together activate expression of two BH3-only factors BCL2L11 and PMAIP1 (also known as BIM and NOXA, respectively) which antagonize the anti-apoptotic BCL2 proteins and lead to the loss of mitochondrial membrane potential [22]. Analysis of our RNA-seq data revealed that DUX4 induced the expression of EGR1 (moderated fold-change of 48), induced the expression of BIMγ, a specific isoform BCL2L11 with an alternative BH3-like domain [23], and increased total BCL2L11 transcripts roughly 2-fold (Fig 2C and S4 Table). The siRNA library used in our screen contained two separate siRNA pools against different splice isoforms of BCL2L11. One of the two pools rescued RD-DUX4i viability (Z-score = 2.01) and contained three siRNAs that targeted only the BIMγ isoform (3 out of 4 siRNAs), whereas the second pool did not rescue (Z-score = -0.59) and did not contain siRNAs that targeted only this isoform (S5F Fig). Together our data indicate that DUX4 expression resulted in decreased degradation of the MYC mRNA and increased abundance of the MYC protein with subsequent activation of components of the MYC-mediated apoptotic pathway, which has been shown to sensitize cells to apoptotic stimuli [24].

### DUX4 expression leads to accumulation of dsRNA and activation of an innate immune response

As noted above, knockdown of *RNASEL*, a gene involved in innate immunity to viral infection, rescued RD cells from DUX4 lethality (DUX4 Z-Score = 3.71) and had minimal effect on the doxycyline induction of the luciferase transgene (Luciferase Z-Score = -0.07). This rescue was validated as on-target based on our deconvolution strategy (S4B Fig) and prompted us to look for other mediators of the cellular antiviral response in our screen. EIF2AK2/PKR is similarly involved in antiviral response and its knockdown marginally rescued from DUX4 toxicity in our original screen (Z-score 1.93), but it passed all other criteria of our validation and filtering process (S4A and S4B Fig). These results led us to further postulate that at least part of DUX4 toxicity might be mediated via triggering an innate (antiviral-like) immune response in expressing cells.

**Fig 3:**
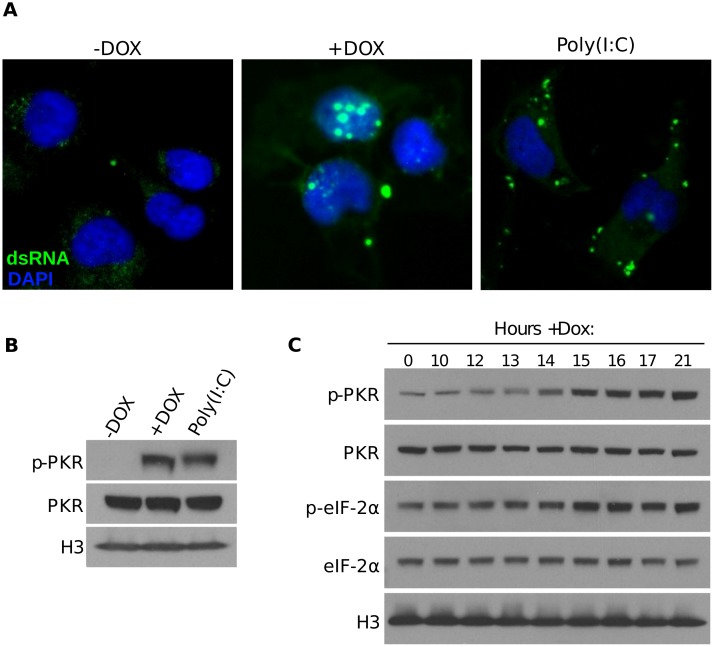
DUX4 induces nuclear dsRNA accumulation and phosphorylation of EIF2AK2/PKR and EIF2S1/eIF-2α. (A) Fluorescence microscopy using the J2 antibody to detect dsRNA in RD-DUX4i cells treated with doxycyline for 16 hours or transfected with poly(I:C). Note that not all nuclei showed dsRNA aggregation, likely due to asynchronous activation of the DUX4 transgene. (B) Western blot of phosphorylated and unphosphorylated PKR in extracts from RD-DUX4i cells treated with doxycyline for 21 hours or transfected with poly(I:C). (C) Western blot in extracts from RD-DUX4i cells treated with doxycyline for the indicated times and using the indicated antibodies.

Both EIF2AK2/PKR and RNASEL are primary responders of the double stranded RNA innate immune response. Because DUX4 leads to the accumulation of normally degraded RNAs, including those destined for nonsense mediated decay [14], activates the expression of RNAs from repetitive regions and retrotransposons [13], and stabilizes some mRNAs (this study), we considered the possibility that DUX4-expressing cells could increase the abundance of aberrant, endogenously formed dsRNAs. In order to test whether DUX4-expressing cells accumulate dsRNAs, we performed immunofluorescence using the J2 antibody, which recognizes dsRNAs, irrespective of the sequence [25]. These experiments revealed that DUX4-expressing cells had strong, nuclear dsRNA foci which were not present in uninduced cells (Fig 3A). The signal observed after transfection of the dsRNA surrogate, poly(I:C), was similar in intensity to DUX4 expressing cells, though occupying a more cytoplasmic compartment (Fig 3A). This signal is not a non-specific property of the J2 antibody as we detected similar focal staining in DUX4-expressing cells using the separate monoclonal dsRNA-recognizing antibody K1 (S6A Fig), nor is it an artifact of the doxycycline induction of transgene mRNA expression as our RD-LUCi line did not have similar nuclear staining after doxycycline treatment (S6B Fig). Immunofluorescence of DUX4-induced RD cells using either the J2 or K1 antibodies showed that approximately 5–10% of the nuclei had obvious dsRNA aggregation at 19 hours post induction, whereas the uninduced cells did not exhibit any detectable nuclear staining.

Accumulation of dsRNAs activates EIF2AK2/PKR via auto-phosphorylation. EIF2AK2/PKR, in turn, phosphorylates eukaryotic initiation factor 2 alpha (EIF2S1/eIF-2α), which is thought to generally inhibit both host and viral gene translation, culminating in cellular apoptosis [26]. To determine whether dsRNA accumulation leads to EIF2AK2/PKR activation in DUX4-expressing cells, we measured phosphorylated EIF2AK2/PKR in our DUX4-expressing RD cells and observed that, following DUX4 expression, EIF2AK2/PKR was phosphorylated to a level that is comparable to activation via transfection of poly(I:C), whereas total EIF2AK2/PKR levels remained unchanged (Fig 3B). To determine whether the EIF2AK2/PKR activation as a result of DUX4 expression has a functional consequence in our cells, we also blotted for EIF2S1/eIF-2α phosphorylation in a time course following doxycycline induction. This experiment showed that both EIF2AK2/PKR and EIF2S1/eIF-2α became phosphorylated approximately 15 hours post doxycycline addition (Fig 3C).

The profound inhibition of the NMD pathway by DUX4 [14] might be the cause of both the increased MYC mRNA and the accumulation of dsRNA because the NMD endonuclease SMG6 has been shown to degrade MYC as a non-canonical NMD target [27] and NMD inhibition leads to dsRNA accumulation in yeast [28]. Therefore, we used siRNAs to knockdown two components of the NMD pathway, UPF1 and SMG6. Knockdown of either NMD component alone or in combination showed a mild, non-significant ~1.1–1.6 fold increase in MYC mRNA and a ~1.6–2.3 fold increase in the inclusion of an NMD-targeted exon in SRSF3, compared to the more than 24-fold increase in these RNAs following DUX4 induction (S7 Fig). Therefore, NMD inhibition through these mechanisms was not sufficient to stabilize the MYC mRNA.

### DUX4 expression in human myoblasts leads to MYC mRNA and dsRNA accumulation with activation of downstream apoptotic pathways

**Fig 4:**
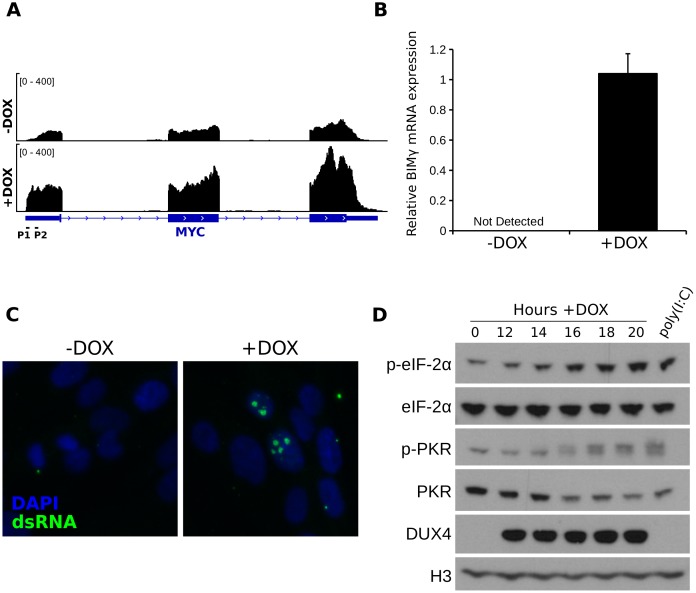
DUX4 induces components of the MYC-mediated apoptotic pathway and the dsRNA response pathway in human muscle cells and FSHD myotubes. (A) Example RNA-seq track showing reads at the MYC locus in MB135-DUX4i cells +/- doxycyline for 14 hours. (B) RT-qPCR using BIMγ isoform specific primers in MB135-DUX4i cells +/- doxycyline for 12 hours. Error bars depict standard deviation of the mean of two independent experiments (C) Confocal microscopy image of MB135-DUX4i cells +/- doxycyline for 20 hours and using the J2 antibody to detect dsRNA. (D) Western blot of MB135-DUX4i cell extracts following DUX4 induction showing phosphorylation of EIF2AK2/PKR and EIF2S1/eIF-2α.

To determine whether DUX4 induces the same MYC-mediated apoptotic pathways and dsRNA toxicity in human myoblasts, we assessed the expression of MYC, components of the MYC-mediated apoptotic pathway, and the dsRNA innate immune response in an immortalized human myoblast cell line with a doxcycycline-inducible DUX4 (MB135-DUX4i). Our previous RNA-seq data generated using MB135-DUX4i cells fourteen hours after DUX4 induction [29] showed an approximate 3.3-fold increase (based on an EdgeR differential expression analysis) in MYC mRNA abundance and, interestingly, a preferential accumulation of transcripts from the P1 promoter (Fig 4A). Similar to RD cells, DUX4 increased the abundance of EGR1 (5.4-fold) and the BH3-only proteins PMAIP1/NOXA (11-fold) and BCL2L11 (4.7-fold) (see Supplemental Table 1 in reference [29]), and induced expression of the BIMγ isoform of BCL2L11 (Fig 4B). Therefore, the same MYC-mediated apoptotic pathways induced by DUX4 in RD cells were similarly induced by DUX4 in human myoblasts.

To determine whether DUX4 expression in human myoblasts activated the same dsRNA pathway as in RD cells, we stained DUX4-expressing myoblasts with the J2 antibody and found strong focal nuclear dsRNA signal, similar to the observed pattern in RD cells (Fig 4C). Expression of DUX4 also led to phosphorylation of EIF2AK2/PKR as well as EIF2S1/eIF-2α in DUX4 expressing myoblast cells, comparable to levels seen after poly(I:C) transfection (Fig 4D).

Similar to RD cells, knockdown of either MYC or RNASEL rescued the MB135-DUX4i human myoblasts from DUX4-induced cell death, which was measured by counting viable cells at two and four days after induction of DUX4 expression (S8A Fig). However, knock-down of either MYC or RNASEL in human myoblasts also caused a delay in the accumulation of DUX4 protein and the activation of a DUX4 target gene, *MBD3L2* (S8B and S8C Fig). Although DUX4 expression was robust at four days following induction (S8B and S8C Fig), a time when the MYC knock-down continued to show strong rescue, these rescue experiments should be interpreted cautiously regarding the individual necessity of MYC or RNASEL for DUX4-induced apoptosis in human myoblasts. In summary, expression of DUX4 in myoblasts, as in RD cells, resulted in increased MYC mRNA and components of the MYC-mediated apoptotic pathways as well as accumulation of dsRNA and activation of the pro-apoptotic dsRNA-sensing innate immune response.

### EIF4A3 aggregates with DUX4-induced dsRNA and EIF4A3 knock-down inhibits NMD

Next, we determined whether the dsRNA aggregates corresponded to known nuclear subdomains or RNA binding proteins by performing immunofluorescence using the K1 dsRNA antibody and costaining with antibodies to promyelocytic leukemia bodies (PML), paraspeckles (NONO), the exon junction complex (EJC) components EIF4A3 and RBM8A/Y14, and TARDBP/TDP-43, which has been shown to aggregate in DUX4-expressing nuclei [30] and may regulate cellular dsRNA accumulation [31]. Components of PML bodies and paraspeckles were not strictly associated with dsRNA staining (S9A Fig), though NONO did form condensations in the nucleus following DUX4 induction, some of which overlapped with dsRNA staining (S9B Fig). TARDBP/TDP-43 did not show a clear association with the dsRNA foci (S9D Fig).

**Fig 5:**
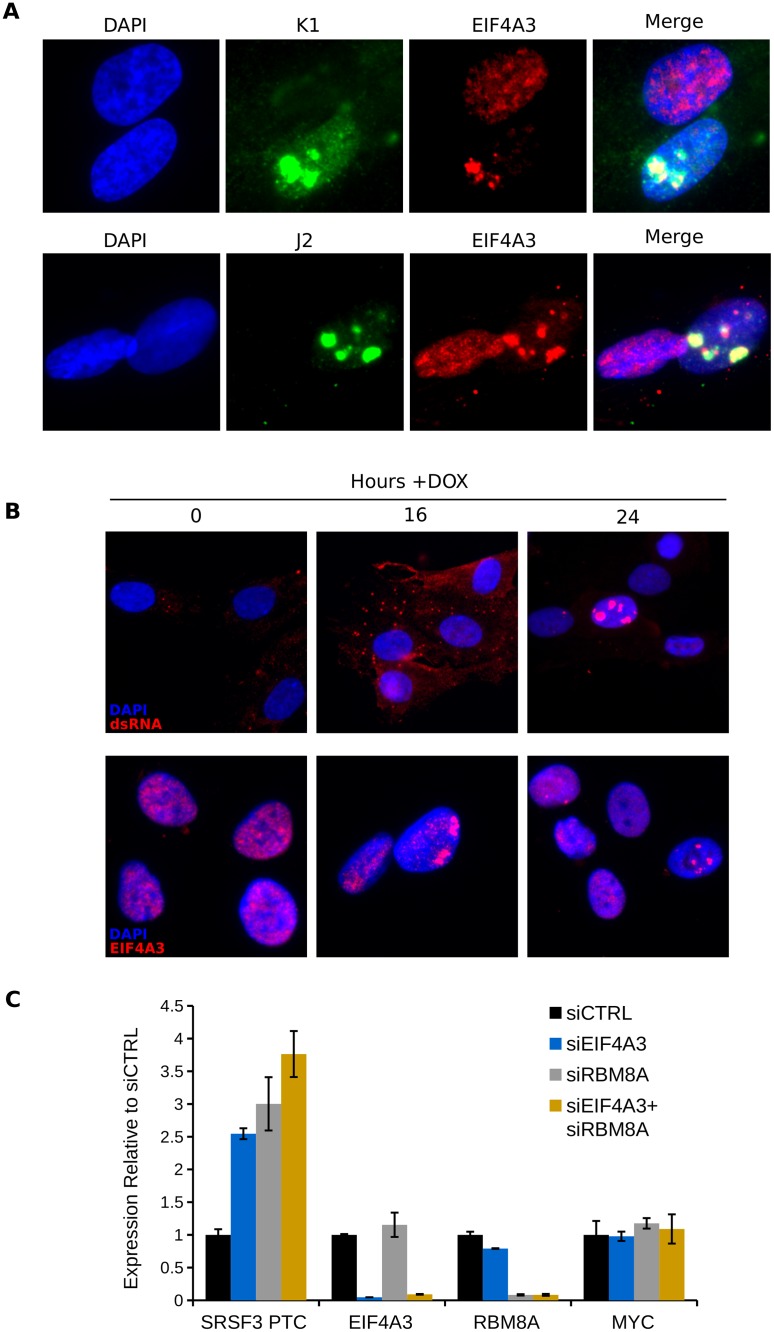
EIF4A3 aggregates with DUX4-induced dsRNAs correlating with inhibition of NMD. (A) Immunofluorescence showing fields of MB135-DUX4i containing nuclei with and without dsRNA aggregates determined using either K1 or J2 antibodies, as indicated, and co-stained against EIF4A3. (B) Time course experiment in MB135-DUX4i cells induced for the indicated period of time and stained against dsRNA (K1) or EIF4A3, as indicated. (C) RT-qPCR using the indicated primers and the corresponding knockdowns, harvested 48 hours after transfection. SRSF3 PTC indicates SRSF3 isoform normally degraded by NMD. Data are normalized to RPL27A and shown relative to the siCTRL condition. Error bars represent the standard deviation of the mean of three replicate wells.

In contrast, DUX4 strongly induced redistribution of EIF4A3 into aggregates that were almost entirely associated with dsRNA foci as determined by double-staining with either the J2 or K1 antibodies (Fig 5A). In many cases DUX4-induced cells with EIF4A3 aggregates appeared to have reduced nuclear staining beyond the aggregates (Fig 5A) suggesting that the majority of EIF4A3 was redistributed to the dsRNA foci, similar to the depletion of MBNL proteins by the mutant nuclear RNA in myotonic dystrophy [32,33]. The other EJC component tested, RBM8A/Y14, showed some association with the dsRNA foci but was not strictly associated with dsRNA aggregates, nor was it depleted from the rest of the nucleus (S9C Fig).

A time course staining cells at 0, 16, or 24 hours following DUX4 induction with antibodies to either dsRNA or EIF4A3 (Fig 5B) showed the initial accumulation of cytoplasmic dsRNA at 16 hours, a time point where EIF2AK2/PKR and EIF2S1/eIF-2α phosphorylation was becoming evident (see Fig 4D). The formation of nuclear dsRNA foci initiated at 16 hours and increased through 24 hours. EIF4A3 nuclear foci were present at 16 hrs and more abundant at 24 hours.

DUX4 expression inhibits NMD and at least part of this inhibition is secondary to decreased UPF1 protein levels [14], and knockdown of UPF1 showed a modest increase in NMD-targeted SRSF3 isoform in RD cells (S7 Fig). Similarly, knockdown of EIF4A3 or RBM8A/Y14 in MB135 cells resulted in a substantial increase (~2.5–3.8 fold) in the NMD-targeted SRSF3 isoform (Fig 5C), suggesting that the nuclear sequestration of EIF4A3 might contribute to NMD inhibition in DUX4-expressing cells. MYC mRNA levels were not affected by EIF4A3 of RBM8A/Y14 knockdown (Fig 5C).

### FSHD cells have increased MYC expression and foci of dsRNA and EIF4A3

Endogenous DUX4 is expressed in only a small percentage of cultured FSHD muscle cells [2]. Therefore, to determine whether DUX4 expression in FSHD cells correlates with higher levels of MYC mRNA, we used an RNA-seq dataset from FSHD cells FACS sorted based on the expression of a DUX4-reporter gene [7]. Based on our previous analysis of this dataset (see Supplemental Table 1 in Jagannathan, *et al* [29]), muscle cells expressing the DUX4-responsive promoter showed an almost 2-fold increase in the level of MYC mRNA (log2 fold-change ~0.9 and adjusted p-value = ~0.003); however, EGR1, PMAIP1/NOXA and BCL2L11 were not significantly upregulated. Therefore, DUX4 expression is associated with higher levels of MYC mRNA in FSHD muscle cells, but the role in activating an apoptotic pathway requires further study.

**Fig 6:**
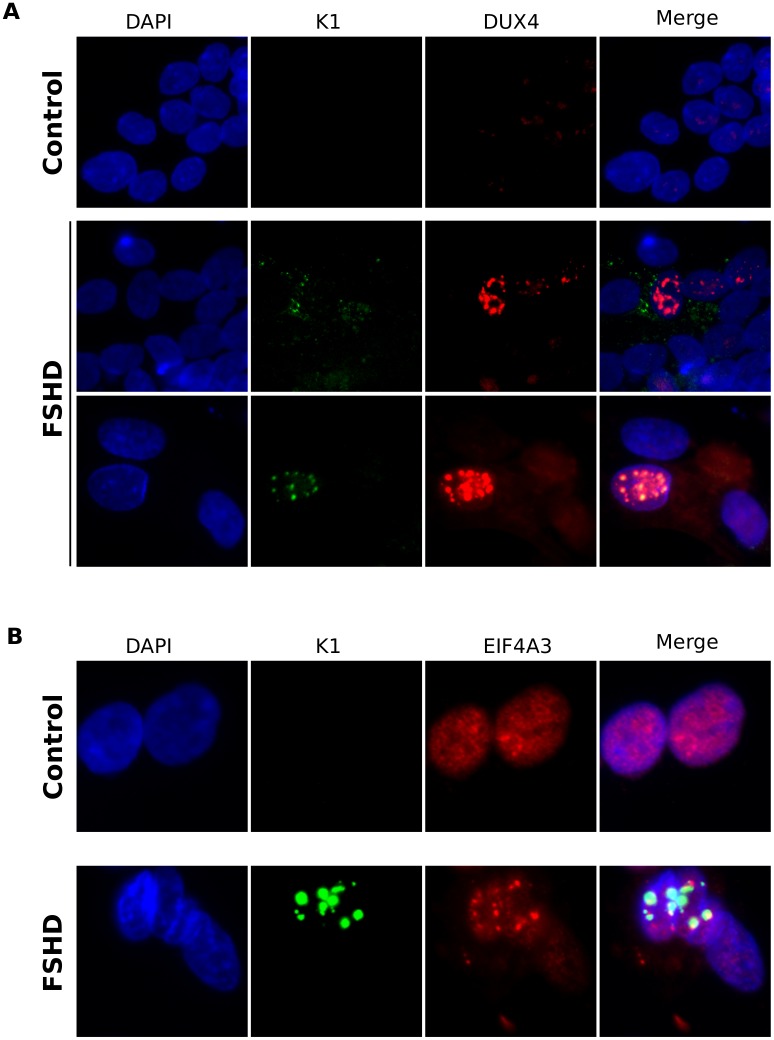
FSHD muscle cells have increased dsRNA and EIF4A3 aggregation. (A) Immunofluorescence showing dsRNA (K1) and DUX4 staining in control (MB135) and FSHD (MB200) muscle cells differentiated for 48 hours. (B) as in (A) but co-stained with K1 and EIF4A3.

Immunofluorescence for dsRNAs in differentiated FSHD muscle cells showed either cytoplasmic or nuclear dsRNA staining in some of the cells with DUX4-positive nuclei but not in a control cell line (Fig 6A). In addition, the nuclear dsRNA foci in FSHD muscle cells were associated with EIF4A3 aggregates that were not present in control muscle cells (Fig 6B). Therefore, DUX4-expressing FSHD muscle cells showed increased levels of MYC mRNA and formation of nuclear foci with dsRNA and EIF4A3 accumulation, indicating that the initial screen identified pathways relevant to FSHD biology, and perhaps pathophysiology.

## Discussion

In this study, an siRNA screen identified that the MYC-mediated apoptoic pathway as well as components of the dsRNA response pathways were involved in DUX4-induced apoptosis in RD cells, and led to the demonstration that DUX4 expression resulted in the stabilization and accumulation of MYC mRNA, the accumulation of nuclear dsRNA, and formation of nuclear EIF4A3 foci. The accumulation of MYC mRNA correlated with higher MYC protein levels and the transcriptional activation of components of the MYC-mediated pro-apoptotic pathway, including EGR1, BCL2L11 and its BIMγ isoform. The accumulation of dsRNA was correlated with the activation of the EIF2AK2/PKR innate immune response and the phosphorylation of EIF2S1/eIF-2α. Inhibiting the expression of individual components of each of these pathways with siRNAs diminished apoptosis in RD cells in response to DUX4. These pathways were also induced by DUX4 in an immortalized human skeletal muscle cell line, with increased MYC RNA and the downstream effectors of MYC-mediated apoptosis (EGR1, PMAIP1/NOXA, and the BIMγ isoform of BCL2L11), as well as the accumulation of dsRNA, EIF2AK2/PKR and EIF2S1/eIF-2α phosphorylation, and nuclear foci consisting of EIF4A3 and dsRNA. Finally, the elevation of MYC mRNA, dsRNA accumulation, and EIF4A3 nuclear foci in FSHD muscle cells suggests that these processes might contribute to FSHD pathophysiology.

Although DUX4 expression caused apoptosis in a P53-dependent manner in some cells [5], we found that P53 was not necessary for apoptosis in either RD cells or human myoblasts. It is possible that different requirements for P53 might be due to differences in the cell type or the mechanism of DUX4 delivery. This would be consistent with the fact that MYC causes apoptosis in a manner that is either P53-dependent or P53-independent, depending on the cellular context [34].

EIF4A3 is a component of the EJC complex that binds the exon-junction of spliced RNAs [35]. Although the mechanism of sequestering EIF4A3 to nuclear foci remains to be determined, the co-localization of the EIF4A3 foci with dsRNA foci suggests that DUX4 induction and/or stabilization of RNAs with dsRNA secondary structure might overwhelm normal nuclear RNA processing and transport, leading to their accumulation as foci with associated RNA binding proteins. The formation of EIF4A3 nuclear aggregates might contribute to the inhibition of NMD following DUX4 expression, because the formation of nuclear aggregates appears to deplete EIF4A3 from the rest of the nucleus and knockdown of EIF4A3 inhibits NMD. In this regard, FSHD might have some parallels with myotonic dystrophy, where the MBNL RNA binding proteins are sequestered in nuclear foci by the repeat expansion in the mutant RNA [33]. However, our prior demonstration that DUX4 expression leads to degradation of the UPF1 protein indicates that the possible sequestration of EIF4A3 is not the only mechanism by which DUX4 might inhibit NMD, and that simply re-expressing EIF4A3 might be unlikely to fully rescue this defect. Further studies will be necessary to determine the identity of the DUX4-induced dsRNAs and the RNAs that mediate EIF4A3 aggregation.

Although it is currently difficult to diagram a clear epistatic model from our results, there are multiple known interactions between MYC, the RNA-mediated innate immune response, and NMD. It has been shown that MYC overexpression can inhibit NMD, leading to ROS-mediated stress and EIF2S1/eIF-2α phosphorylation [36], both of which are associated with DUX4 expression ([6] and this study). Thus the elevated MYC in DUX4 expressing cells might also contribute to NMD inhibition, feeding forward to further enhance levels of DUX4, which is also an NMD target [14]. Inhibition of NMD might also lead to the accumulation of dsRNAs. In yeast, NMD controls the accumulation of antisense long non-coding derived dsRNAs [28]. It is therefore possible that the profound inhibition of NMD by DUX4 might similarly result in dsRNA accumulation, either from constitutively expressed RNAs or the many repetitive RNA families and LTR-containing ERVs transcriptionally activated by DUX4 [13,37]. Elevated levels of dsRNAs would then lead to an innate immune response, as has been observed in cancer cells that reactivate LTR-containing ERVs after DNA methylation inhibitor treatment [38,39]. While we observed phosphorylation of EIF2AK2/PKR, which occurs upon binding dsRNA ligand [40], we did not see a robust interferon response (based on our RNA-seq data). However, DUX4 has been shown to inhibit components of the innate immune response pathway, at least in part through upregulation of DEFB103 [12], suggesting that the activation of this pathway by DUX4 might have a normal developmental role.

The apoptotic pathways induced by DUX4 in RD cells and human myoblasts might give insight into its normal role in development. DUX4 is normally expressed in the testis, likely in the early germline cells such as the spermatogonia and primary spermatocytes, and has recently been reported to be expressed in the thymus, both areas of active developmental apoptosis [2,3]. It is possible that one normal function for DUX4 is to elicit cellular apoptosis, and indeed the BIMγ isoform of BCL2L11 that we showed is induced by DUX4 in skeletal muscle is also expressed in the testis [23]. However, the broad transcriptional program activated by DUX4 suggests a role in stem cell function beyond apoptosis, and apoptosis might occur in a cell-context dependent manner. In other words, cells that normally express DUX4 might be primed for apoptosis but also resistant in the correct developmental context. As mentioned above, DUX4 expression blocks the RNA-induced innate immune response to viral infection [12], which might constitute a mechanism for protecting DUX4-expressing cells from toxicity in some normal developmental contexts compared to the expression of DUX4 in skeletal muscle cells. We speculate that DUX4 expression, which leads to elevated MYC levels, might afford an accompanying growth or fitness advantage in the developmental context where cells are competing for resources and survival. This notion is further supported by the fact that the MYC-mediated competitive advantage depends on the innate immune response pathway [41]. This normal process of cell competition is believed to control developmental size and eliminate cells with weaker attributes. Furthermore, the competitive advantage conferred by MYC might also be used in cancers for clonal expansion [42]. In this regard, it is interesting to note that DUX4 expression has recently been shown to be a causal factor in a subset of B-cell leukemias [43,44]. Further work will be necessary to determine how the DUX4-mediated modulation of MYC levels and RNA accumulation might contribute to aspects of development and the pathophysiology of FSHD.

## Materials and methods

### Ethics statement

This study used pre-existing de-identified human cell lines from approved repositories and was determined to not be Human Subjects Research by the FHCRC Institutional Review Board. The commonly used RD rhabdomyosarcoma cell line was obtained from the ATCC (www.atcc.org). Myoblast samples were obtained from the Fields center at the University of Rochester (https://www.urmc.rochester.edu/fields-center.aspx) and patients gave informed written consent prior to sample use. For the use of third party data, no additional ethics approval was required and original ethics approval can be viewed in Jagannathan, *et al* [29] and Rickard, *et al* [7].

### Cell culture

RD-DUX4i cells were grown in DMEM (Gibco) supplemented with 10% FBS (Hyclone), 1% penicillin/streptomycin (Gibco) and 2.0 μg/ml puromycin (Sigma). The DUX4 transgene was induced with 1.0 μg/ml of doxycycline hyclate (Sigma). Unaffected (MB135) and FSHD (MB200) immortalized human myoblasts were cultured in F10 medium (Gibco/Life Technologies) supplemented with 20% FBS and 1% penicillin/streptomycin as well as 10 ng/ml recombinant human FGF (Promega) and 1 μM dexamethasone (Sigma). Human myoblasts were differentiated by culturing in knockout serum replacement medium (Gibco/Life Technologies).

### Cloning, virus production and monoclonal cell line isolation

The DUX4 and firefly luciferase genes were subcloned into the NheI and SalI sites of the pCW57.1 vector (Addgene plasmid #41393). Lentiviral particles were produced by co-transfecting 293T cells with the pCW57.1 vector, pMD2.G (Addgene plasmid #12259) and psPAX2 (Addgene plasmid #12260) using Lipofectamine 2000 (ThermoFisher) and following the manufacturer's instructions. Human RD cells were transduced and selected using 2.0 μg/ml puromycin (Sigma) at a sufficiently low multiplicity of infection to allow for clonal lines to be isolated using cloning cylinders. The MB135-DUX4i monoclonal cell line was produced as described in [29].

### Caspase activity assay

RD-DUX4i cells were seeded in a 96-well plate, induced with doxycycline for 48 hours, then assayed for caspase 3/7 activity using Caspase-Glo Assay Technology (Promega) according to the manufacturer’s instructions.

### Cell viability and luciferase assays

RD-DUX4i cells were reverse transfected using LipofectamineRNAiMAX (Invitrogen) reagent in 96-well plates with 6,000 cells/well and 1.25 pmol/well of siRNAs (flexitube, Qiagen). Cells were induced with doxycycline at 1.0 μg/ml for 48–96 hours prior to assaying, depending on the experiment. Viability was assessed using CellTiter-Glo assay (Promega) per the manufacturer’s instructions. Luciferase activity was assessed using ONE-Glo EX Luciferase Assay System (Promega), per the manufacturer’s instructions.

### MB135 human myoblast *TP53* CRISPR knockout

Duplexed oligonucleotide containing the guide RNA sequence against *TP53* was cloned into lentiCRISPRv2 plasmid (Addgene plasmid #52961), essentially using the Zhang lab protocol [45]. We used the sequence: CGCTATCTGAGCAGCGCTCA for targeting purposes. Cells were transduced with lentiviral particles produced as above, selected with 2.0 μg/ml puromycin and then clonally reseeded such that isolated colonies could be picked. Mutations were subsequently identified by Sanger sequencing Topo-cloned (ThermoFisher) PCR amplicons which flanked the targeted region. Cells were treated with 10.0 μM of actinomycin D (Sigma) in DMSO to test induction of P53.

### siRNA library and screen parameters

The Human Druggable Genome siRNA Set V4.0 was purchased from QIAGEN and contains 27,844 unique siRNAs multiplexed to target 6,961 genes in “druggable” functional groups such as receptors, nucleic acid binders, kinases, transcription factors, and signaling molecules. The master library was diluted with nuclease-free water (HyClone) into a 96-well parent library at a concentration of 0.5 pmol/μl and then converted to a 384-well child library set. All library manipulations were performed with a Beckman Biomek FX liquid handling robot using filtered sterile tips (ART BioRobitix). 2.5 μl of 0.125 pmol/μl siRNA solution was added to each of three replicate 384-well assay plates (ThermoFisher Scientific). Opti-MEM (Gibco) and RNAiMAX transfection reagent (ThermoFisher Scientific) were combined at 3.0 μl/ml RNAiMAX to Opti-MEM and subsequently distributed at 12 μl/well. 25.0 μl of cells at a concentration of 60,000 cells/ml of growth medium was then added to each well and plates were incubated for 24 hours at 37°C. Medium was then aspirated using a BioTek ELx405 Select cell washer and doxycycline-containing growth medium was added at 25.0 μl/well using the BioTek Micro Flo Select and incubated for an additional 72 hours. CellTiter-Glo reagent was added at 20.0 μl/well and luminescence was read on a microplate reader (Perkin Elmer EnVision 2104). Analysis of data was performed using the CellHTS2 Bioconductor package [46] and was normalized by using the median method. Protein interaction network analysis was performed using ConsensusPathDB software considering only high-confidence protein interactions [47].

### RNA half-life experiments

RD-DUX4i cells were pre-incubated in the presence of doxycycline for 8 hours prior to treatment with DRB (Sigma) at 75 μM for up to two hours and levels were quantified using real time qPCR.

### RNA isolation and real time qPCR

Cells were seeded at 66 x10^3^ cells/well in 12-well plates. RNA was isolated using TRIzol reagent (ThermoFisher Scientific) according to manufacturer’s instructions. Isolated RNA was treated with DNaseI (ThermoFisher Scientific), heat inactivated and reverse transcribed into cDNA using Superscript III (ThermoFisherScientific) following the manufacturer’s protocol. Real time qPCR was performed using SYBR green reagent (ThermoFisher Scientific) for quantification.

### RNA-seq library preparation and data analysis

RNA-seq of RD-DUX4i cells was performed on RNA samples collected from 3 experimental replicates induced for 16–18 hours with doxycycline. Illumina TruSeq libraries were prepared with 500 ng total RNA per sample using the standard library preparation protocol. 100 bp single-end sequencing was performed using the HiSeq 2500 platform by the FHCRC Genomics facility. Base calling was performed with Real Time Analysis software version 1.18.66.3. Raw reads were aligned to UCSC hg38 using Bowtie2 version 2.2.6 and Tophat version 2.1.0 [48]. Gene counts were calculated using the Gencode version 22 annotation file (obtained from the UCSC genome browser) and the GenomicAlignments version 3.3 Bioconductor package [49] in R version 3.4.0 using the intersection-strict mode. Differential expression analysis was performed using DESeq2 version 1.12.4 [50]. FPKM reads were calculated using a custom R script. Processed data were visualized using IGV software [51]. Raw and processed data have been deposited onto the NCBI Gene Expression Omnibus under the accession number GSE87495.

### Western blotting

Reduced and boiled samples (typically 10–20 μg total protein per assay) were run on NuPage 4–12% precast polyacrylamide gels (Life Technologies) and transferred to PVDF membrane (Life Technologies). After blocking in 5% milk in PBST, the membrane was incubated with appropriate antibodies (described below) in block solution overnight at 4°C. Membranes were then incubated with appropriate HPRT-conjugated secondary antibodies in block solution for one hour at room temperature and chemiluminescent substrate (ThermoFisher Scientific) was used for detection. Densitometry, when performed, was achieved using ImageJ software [52].

### dsRNA immunofluorescence

For all experiments involving RD cells, the cells were fixed with 4% paraformaldehyde (Electron Microscopy Sciences) in PBS for seven minutes at room temperature prior to permeabilization with 0.5% Triton X-100 (Sigma) in PBS. Cells were then incubated with 1% BSA in PBS block solution for 30 minutes at room temperature and then incubated overnight with J2 or K1 antibody at a concentration of 2 μg/ml at 4°C. Appropriate FITC- or TRITC-conjugated secondary antibodies (Jackson ImmunoResearch) and DAPI stain (Sigma) were then used prior to visualization. For the human myoblast experiment, conditions were essentially the same, except that cells were fixed with 2% paraformaldehyde and co-incubated overnight with J2 or K1 and anti-DUX4 (E5-5) antibody. Cells were imaged on a Zeiss AxioPhot or, if indicated, a Leica TCS SP5 II confocal microscope and channel merging was performed using ImageJ software.

### Chromatin immunoprecipitation

Cross-linked ChIP coupled with MNase digestion was performed in triplicate on RD-DUX4i cells post doxycycline induction as described in [53] with two minor modifications: decreased MNase concentration (15 units of Worthington MNase incubated at 37°C for 12 min) and greater sonication intensity (4 pulses at 30% amplitude, 15 seconds per pulse with 1 min rest between pulses). Briefly, 10 million RD-DUX4i cells were harvested per sample, 12.5 hours after the induction of DUX4. After crosslinking with 1% formaldehyde for 10 min, samples were treated with MNase followed by sonication. 10 percent of the soluble chromatin from each sample was reserved as input and the remainder of each sample was divided into three equal aliquots for the mock, 8WG16, and Ser2P ChIPs. Pol II antibodies were pre-bound to Protein G dynabeads (ThermoFisher Scientific) for 4 hours at room temperature, according to the manufacturer's directions and incubated with chromatin overnight.

### Antibodies (name, company, batch if available)

Histone H3, Abcam ab1791; MBD3L2, Abcam ab107999, lot GR126890-2; MYC (9E10), Santa Cruz Biotech SC-40; dsRNA, SciCons J2, batch J2-1505; dsRNA, SciCons K1, batch K1-1502; PKR, Cell Signaling D7F7, lot 1; phosphorylated PKR, Abcam ab32036, lot GR155191-6; eIF-2α, Santa Cruz Biotech SC-11386; phosphorylated eIF-2α, Abcam ab32157; 8WG16 UnphosphorylatedPolII, Abcam ab817, lot GR153063-402; Serine2 phosphorylated PolII, Abcam ab5095, lot GR271493-1; PML, Santa Cruz Biotech SC-5621, lot B1216; eIF4A3, Abcam ab180573, lot GR148643-3; nmt55/p54nrb (NONO), Abcam ab133574, lot GR97976-7; Y14 (RBM8A), Abcam ab181038 lot GR152694-1; TDP43, Proteintech 1078-2-AP; p53 (DO-1), Santa Cruz Biotech SC-126, lot l2316; rabbit monoclonal antibodies against DUX4 (E14-3 and E5-5) were produced in collaboration with Epitomics and are described elsewhere [54].

### Primers (name and sequence)

MYC_5prime_chip_1F: GAGGCTATTCTGCCCATTTG
MYC_5prime_chip_1R: TCGGTGCTTACCTGGTTTTC
Chr2_neg_F1: TCACACTTCAGGAAAGCCCC
Chr2_neg_R1: GCAGGCCAGTTTGGGAAAAG
Leutx_5’_F1: CTGCAGCACACAGCTGATCG
Leutx_5’_R1: CTTGCCTTCGCCCAACTTAC
C-MYC-1F: TACAACACCCGAGCAAGGAC
C-MYC-1R: AGCTAACGTTGAGGGGCATC
JUN_1F: GGAGACAAGTGGCAGAGTCC
JUN_1R: CCAAGTTCAACAACCGGTGC
CITED2_1F: AAAGGGAACGGCTCCGAATCTG
CITED2_1R: GCCATCATATGGTCTGCCATTTC
LG185-F (Zscan4): TGGAAATCAAGTGGCAAAAA
LG186-R (Zscan4): CTGCATGTGGACGTGGAC
hDUX4_ex2-3 F2: CGGAGAACTGCCATTCTTTC
hDUX4_ex2-3 R2: CAGCCAGAATTTCACGGAAG
Bim_exon2_F_2: GGGCCCCTACCTCCCTAC
Bim_exon5_R_1: TGGTGGTGGCCATACAAATC
RPL27-1L: GCAAGAAGAAGATCGCCAAG
RPL27-1R: TCCAAGGGGATATCCACAGA
18s_rRNA_F: GTAACCCGTTGAACCCCATT
18s_rRNA_R: CCATCCAATCGGTAGTAGCG
JZ69-smg6-fwd: TGCTTACTTAAGGAGTCCGCC
JZ70-smg6-rev: TCAGGTCCGGGACAAAGGAA
RKB_321 (SRSF3 PTC): GGGTGGTGAGAAGAGACATGA
RKB_322 (SRSF3 PTC): CTTGGAGATCTGCGACGAG
RKB_328 (UPF1): CAGCTCGCAGACTCTCACTTT
RKB_329 (UPF1): TGCGTCTGGCTAGGAAGAGT
pCW57.1_DS2F (transgene): CACCACCACCACCACAAGG
pCW57.1_DS2R (transgene): GAACGGACGTGAAGAATGTG
JZRT_17-RNASEL-1F: TGAGGGACTGTCTGAGTGACC
JZRT_18-RNASEL-1R: TCAGATTTTCGTGTTTTGATGTCGG
JZ80-EIF4A3-1F: TGGCTCCCACAAGAGAGTTG
JZ80-EIF4A3-1R: CTTCCTGATGTCCTCGCCAA
JZ78-RBM8A-1F: GCGAAGATTTCGCCATGGAT
JZ79-RBM8A-1R: TCATAATCCTCACGCATCCGC

## Supporting information

S1 Fig DUX4 induces cell death in *TP53* deficient cells. (A) Caspase 3/7 activity assay (Caspase-Glo) 48 hours following doxycycline induction in RD-DUX4i cells. Error bars represent the standard deviation of the mean of three replicate wells. (B) CellTiter-Glo viability assay of parental (unmodified) MB135 and *TP53* knockout MB135 (MB135 P53-/-) immortalized human myoblast cell lines transduced with the indicated volume of DUX4 lentiviral expression vector. Note that, because DUX4 induces cell death, it is not possible to conventionally titer the virus. (C) Representative Sanger sequencing results of knockout cell line depicting the region of *TP53* target site where an indel was induced by non-homologous end joining from CRISPR/Cas9 directed cleavage. Two of 14 topo-cloned PCR amplicons had a two nucleotide insertion whereas the other 12 amplicons had a single nucleotide insertion at the cleavage site and no wild-type sequences were observed indicating that our *TP53* knockout MB135 cell line has a frameshift mutation in both alleles. (D) Western blot showing P53 levels in WT (parental) and *TP53* knockout MB135 cell line. P53 was induced by actinomycin D (ActD) which was added to growth medium for 24 hours prior to harvesting and serves as a positive control for detecting the endogenous levels of P53.
(TIF)

S2 Fig siRNA screen identifies targets that diminish DUX4 toxicity in RD cells. (A) Schematic of the all-in-one pCW57.1 inducible lentiviral system used to express DUX4. Explanation of abbreviations used: TRE: tetracycline response element; CDS: coding DNA sequence; hPGK: human phosphoglycerate kinase 1 promoter; PuroR-T2A-rtTA: co-expressed puromycin N-acetyltransferase resistance gene, 2A peptide which yields separate translation of the tetracycline controlled transactivator. (B) Phase contrast images showing morphology of RD-DUX4i cells 24 hours +/- doxycyline. (C) CellTiter-Glo (ATP-based) assay 48 hours +/- doxycyline as a measure of cell viability. Data are relative to the “Dox-” condition. Error bars represent the standard deviation of the mean of three replicate wells. (D) Schematic showing optimized parameters used for the full scale siRNA screen. Briefly, cells were transfected in multi-well plates for 24 hours and subsequently induced to express DUX4 for 72 hours before cell viability was recorded using CellTiter-Glo reagent. (E) Plot ranking all individual siRNA targets from the siRNA screen. The mean of three triplicate wells (large points) and minimum and maximum values of triplicate wells (smaller points above and below) are shown. Note that DUX4-1 siRNA was more robust at knocking down the DUX4 transgene than DUX4-2 siRNA (see also S3B Fig).
(TIF)

S3 Fig Optimization and network analysis of the siRNA screen. (A) CellTiter-Glo viability assay depicting an example of our strategy used to optimize parameters for the full-scale siRNA screen. In this example we varied cell number and dose of doxycyline (concentration in ng/ml). Error bars represent the standard deviation of the mean of three replicate wells. (B) Western blot of inducible DUX4 transgene expression 24 hours following indicated siRNA transfection and subsequent 5 hour induction. (C) ConsensusPathDB induced network module analysis of protein-protein interactions from |Z-score| > 2.0 of unfiltered screen results.
(TIF)

S4 Fig Validation, deconvolution, and synergy screens of siRNA pools. (A) CellTiter-Glo viability assay of select rescuing targets from RD-DUX4i siRNA screen following transfection of indicated siRNA pools in order to determine reproducibility of the original experiment. Viability is shown relative to the siCTRL condition. (B) Deconvolution of pools as in (A). The red dotted line is set at 1.0 as a reference. (C) Viability assay testing pooling of 'non-rescuing' siRNAs from (B) in order to determine whether these siRNAs could 'synergize' or if the response was dominated by a single siRNA (likely off-target result). (D) RD-LUCi cells were treated with siRNAs for 24 hours and induced with doxycycline prior to reading luminescence of luciferase transgene. Error bars in all graphs represent the standard deviation of three replicate wells. (E) Immunofluorescence of DUX4 in RD cells that were transfected with the indicated siRNAs and, after 24 hours, transduced with lenti-DUX4 (pRRLSIN vector backbone with a human PGK promoter driving DUX4 expression). Images were taken 42 hours following DUX4 transduction, when clear viability differences between knockdown conditions were evident. Note that siMYC appeared to have no clear effect on either nuclear localization or overall expression of DUX4 compared to the control knockdown. (F) Western blot showing DUX4 and MYC protein levels following the indicated knockdowns at 18 hours after transduction of lenti-DUX4. (G) CellTiter-Glo viability assay following the indicated knockdowns at 48 hours after transduction of lenti-DUX4.
(TIF)

S5 Fig Determination of DUX4 binding and activation of MYC, RNA stabilization, and the shorter BIMγ isoform of BCL2L11. (A) Track showing RNA-seq or ChIP-seq reads mapped 22kb at and surrounding the MYC locus. Note that there is no apparent DUX4 occupancy near the canonical MYC promoter nor elsewhere. (B) RT-qPCR data of MYC and ZSCAN4 (a direct transcriptional target of DUX4) following doxycyline induction in RD-DUX4i cells. (C) Western blot for protein half-life experiment of MYC in RD-DUX4i cells. Cells were treated with or without doxycyline for 8 hours prior to the addition of the translation inhibitor, cyclohexamide (CHX). MG132, a proteosomal inhibitor, was included as a positive control and added during CHX addition. Densitometry was used to estimate relative protein levels compared to the zero-hour time point and data were fitted onto a semi-log plot in order to estimate the half-life of each condition. (D) ChIP-qPCR of unphosphorylated (8WG16) and Serine-2 phosphorylated forms of RNA polymerase II. The “negative locus” is a primer-set with no known annotated transcripts and serves as a negative control. Error bars represent the standard deviation of three replicate ChIP experiments. (E) mRNA half-life experiment as in Fig 2B. (F) RNA-seq track showing the location of the rescuing and non-rescuing BCL2L11 (BIM) pool of siRNAs in RD cells +/- doxycycline. The shorter splice form induced by DUX4 is the BIMγ isoform of BCL2L11 and the rescuing pool has three siRNA that specifically target this isoform.
(TIF)

S6 Fig dsRNA in RD cells expressing DUX4. (A) Confocal microscopy immunofluorescence of RD-DUX4i cells +/- doxycyline using the K1 antibody to verify the presence of dsRNAs in DUX4 expressing cells. (B) Confocal microscopy immunofluorescence of RD-LUCi and RD-DUX4i cells with doxycycline and stained with the J2 antibody. Note that, based on previous observations, we believe induction of DUX4 occurs asynchronously in the cell population using the doxycycline-inducible system, explaining why there is not universal nuclear dsRNA positivity at the time point assayed.
(TIF)

S7 Fig siRNA knockown of NMD factors modestly elevates MYC and a canonical NMD target. RT-qPCR data on RD-DUX4i cells either induced with doxycycline (Dox+) or uninduced in the presence of the indicated siRNA and using the primer sets annotated on the X-axis. Error bars represent the standard deviation of three separate experiments.
(TIF)

S8 Fig Knockdown of RNASEL and MYC rescue human myoblasts from DUX4 toxicity, but delay transgene expression. (A) Bright field images showing MB135-DUX4i cell morphology after DUX4 expression at 2 and 4 days post-induction. The ATP-based CellTiter-Glo assay gave unusually high readings in control myoblast cells for unknown reasons and thus we used viable cell counts as an alternative. (B) Western blot showing a DUX4 target gene, MBD3L2, and DUX4 transgene expression in MB135-DUX4i cells at the indicated time-points after induction. (C) RT-qPCR to confirm knockdown of target mRNAs. Data are normalized to RPL27A and shown relative to the siCTRL condition. Error bars represent the standard deviation of the mean of three replicate wells.
(TIF)

S9 Fig Immunofluorescence panel of DUX4-induced dsRNAs with PML, NONO, RBM8A, and TDP-43. MB135-DUX4i cells were induced for 24 hours with doxycycline prior to co-staining with antibodies against dsRNA (K1) and (A) PML. (B) NONO (paraspeckles). (C) RBM8A. (D) TDP-43, none of which show exclusive overlap with dsRNA accumulation.
(TIF)

S1 Table RD-DUX4i druggable genome screen. (XLS)

S2 Table RD-LUCi secondary screen. (XLS)

S3 Table All targets with Z-score > 2.0 from RD-DUX4i screen with RNA-seq FPKM counts. (XLS)

S4 Table RD-DUX4i RNA-seq DESeq2 analysis. (XLS)
