# Supplementary figures and images for: DUX4-induced dsRNA and MYC mRNA stabilization activate apoptotic pathways in human cell models of facioscapulohumeral dystrophy
Source: PLoS Genet. 2017 Mar 8;13(3):e1006658. doi: 10.1371/journal.pgen.1006658 (PMC5362247; doi:10.1371/journal.pgen.1006658)

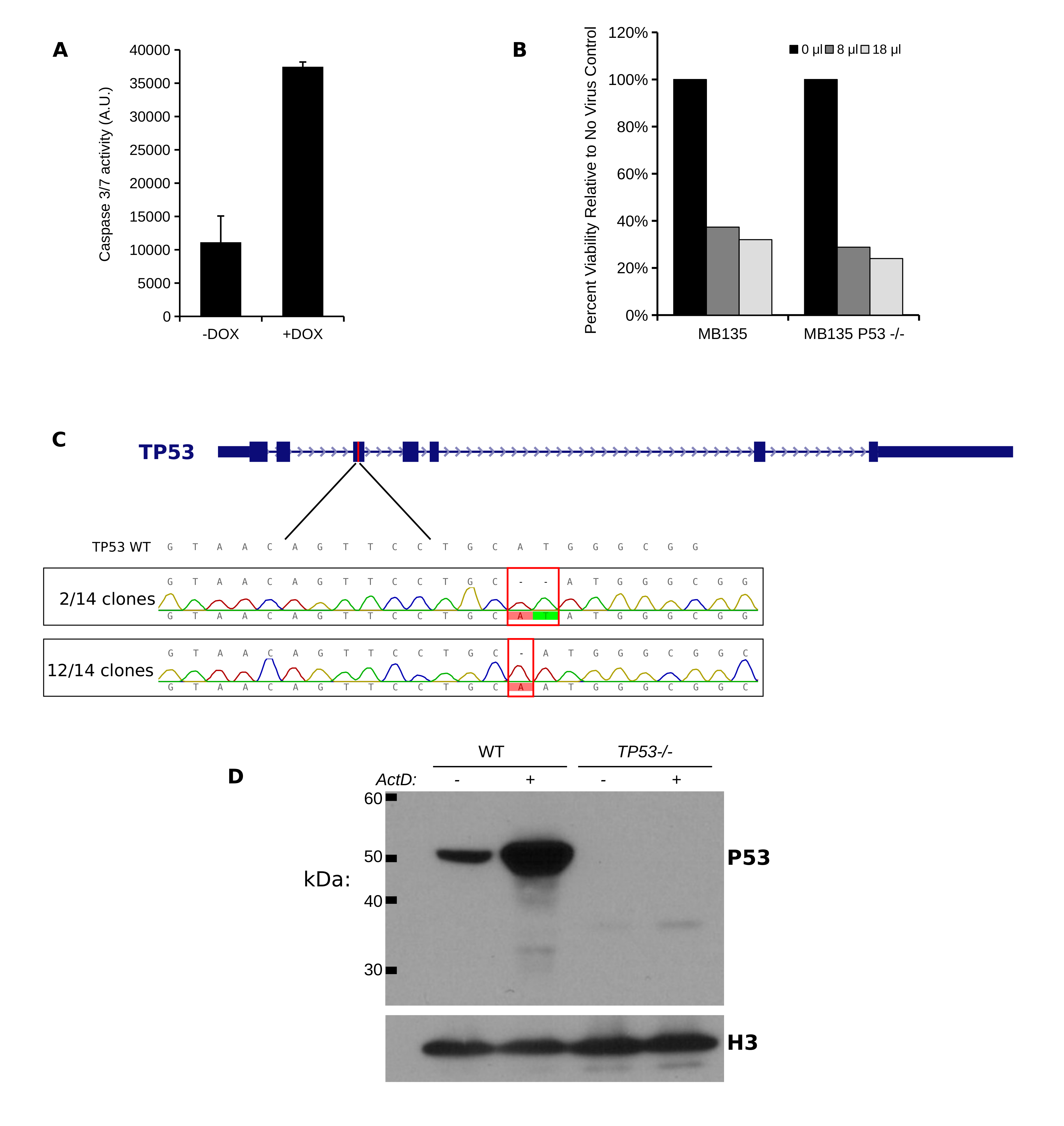

Supplement: S1 Fig — (A) Caspase 3/7 activity assay (Caspase-Glo) 48 hours following doxycycline induction in RD-DUX4i cells. Error bars represent the standard deviation of the mean of three replicate wells. (B) CellTiter-Glo viability assay of parental (unmodified) MB135 and TP53 knockout MB135 (MB135 P53-/-) immortalized human myoblast cell lines transduced with the indicated volume of DUX4 lentiviral expression vector. Note that, because DUX4 induces cell death, it is not possible to conventionally titer the virus. (C) Representative Sanger sequencing results of knockout cell line depicting the region of TP53 target site where an indel was induced by non-homologous end joining from CRISPR/Cas9 directed cleavage. Two of 14 topo-cloned PCR amplicons had a two nucleotide insertion whereas the other 12 amplicons had a single nucleotide insertion at the cleavage site and no wild-type sequences were observed indicating that our TP53 knockout MB135 cell line has a frameshift mutation in both alleles. (D) Western blot showing P53 levels in WT (parental) and TP53 knockout MB135 cell line. P53 was induced by actinomycin D (ActD) which was added to growth medium for 24 hours prior to harvesting and serves as a positive control for detecting the endogenous levels of P53. (TIF) [file pgen.1006658.s001.tif]

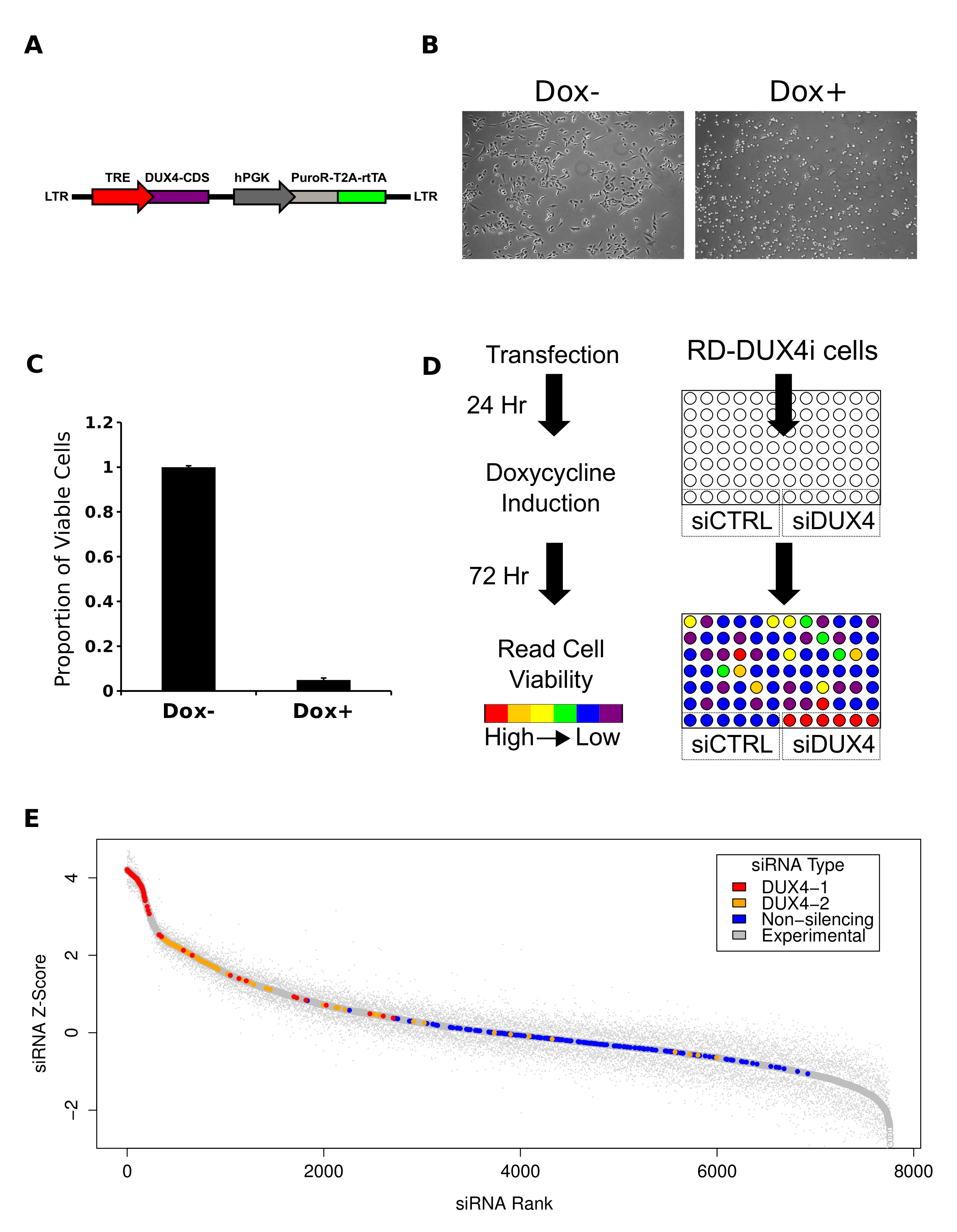

Supplement: S2 Fig — (A) Schematic of the all-in-one pCW57.1 inducible lentiviral system used to express DUX4. Explanation of abbreviations used: TRE: tetracycline response element; CDS: coding DNA sequence; hPGK: human phosphoglycerate kinase 1 promoter; PuroR-T2A-rtTA: co-expressed puromycin N-acetyltransferase resistance gene, 2A peptide which yields separate translation of the tetracycline controlled transactivator. (B) Phase contrast images showing morphology of RD-DUX4i cells 24 hours +/- doxycyline. (C) CellTiter-Glo (ATP-based) assay 48 hours +/- doxycyline as a measure of cell viability. Data are relative to the “Dox-” condition. Error bars represent the standard deviation of the mean of three replicate wells. (D) Schematic showing optimized parameters used for the full scale siRNA screen. Briefly, cells were transfected in multi-well plates for 24 hours and subsequently induced to express DUX4 for 72 hours before cell viability was recorded using CellTiter-Glo reagent. (E) Plot ranking all individual siRNA targets from the siRNA screen. The mean of three triplicate wells (large points) and minimum and maximum values of triplicate wells (smaller points above and below) are shown. Note that DUX4-1 siRNA was more robust at knocking down the DUX4 transgene than DUX4-2 siRNA (see also S3B Fig). (TIF) [file pgen.1006658.s002.tif]

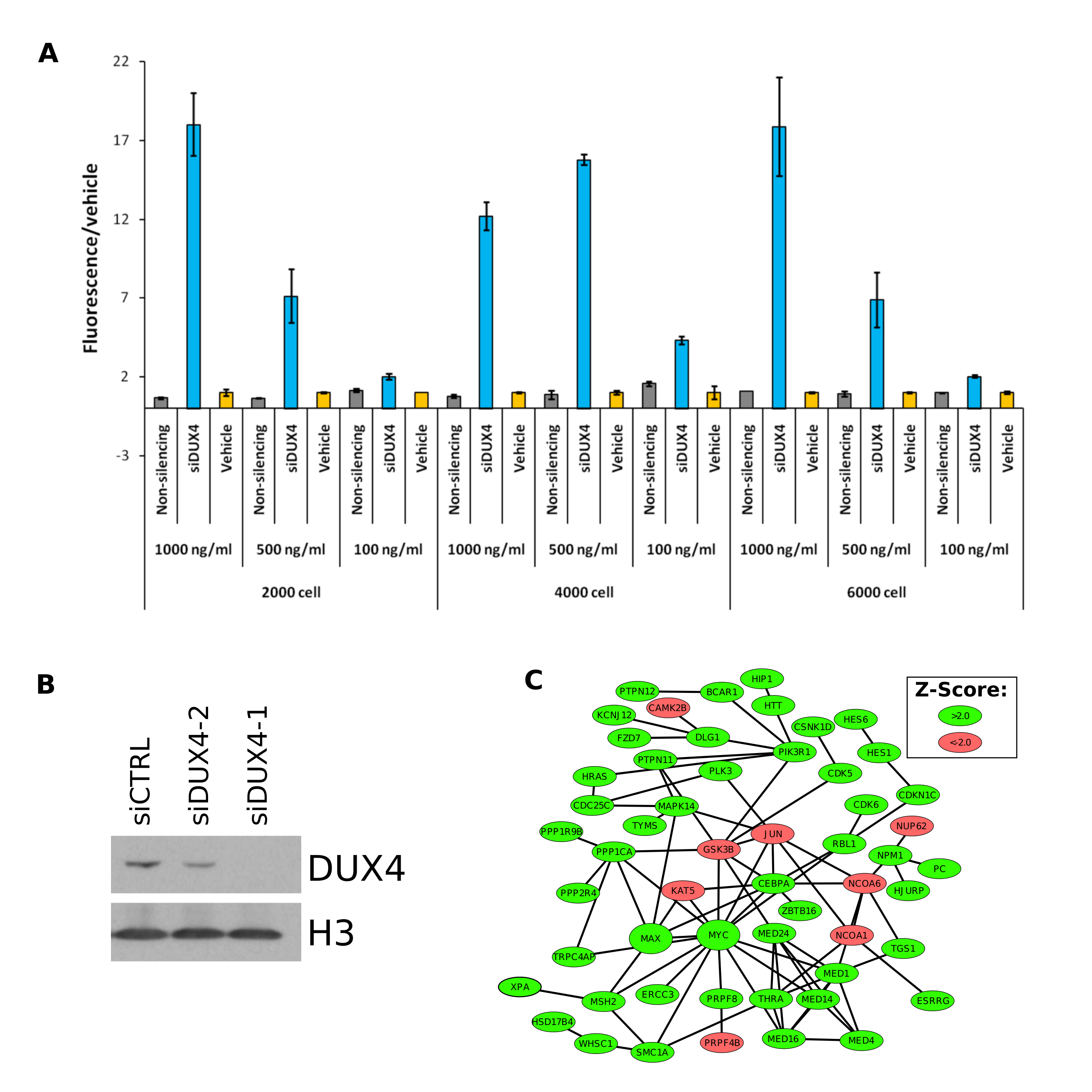

Supplement: S3 Fig — (A) CellTiter-Glo viability assay depicting an example of our strategy used to optimize parameters for the full-scale siRNA screen. In this example we varied cell number and dose of doxycyline (concentration in ng/ml). Error bars represent the standard deviation of the mean of three replicate wells. (B) Western blot of inducible DUX4 transgene expression 24 hours following indicated siRNA transfection and subsequent 5 hour induction. (C) ConsensusPathDB induced network module analysis of protein-protein interactions from |Z-score| > 2.0 of unfiltered screen results. (TIF) [file pgen.1006658.s003.tif]

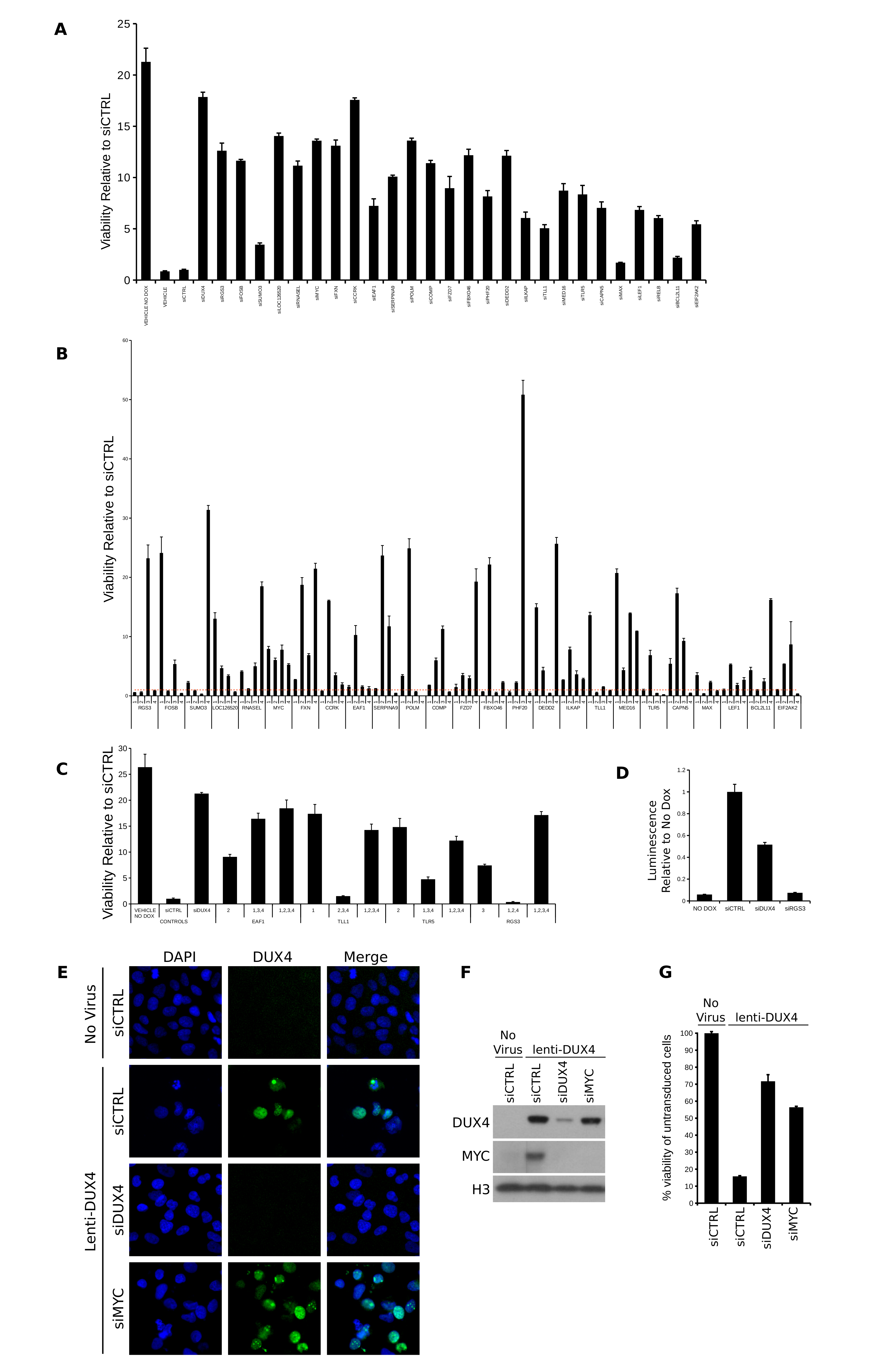

Supplement: S4 Fig — (A) CellTiter-Glo viability assay of select rescuing targets from RD-DUX4i siRNA screen following transfection of indicated siRNA pools in order to determine reproducibility of the original experiment. Viability is shown relative to the siCTRL condition. (B) Deconvolution of pools as in (A). The red dotted line is set at 1.0 as a reference. (C) Viability assay testing pooling of 'non-rescuing' siRNAs from (B) in order to determine whether these siRNAs could 'synergize' or if the response was dominated by a single siRNA (likely off-target result). (D) RD-LUCi cells were treated with siRNAs for 24 hours and induced with doxycycline prior to reading luminescence of luciferase transgene. Error bars in all graphs represent the standard deviation of three replicate wells. (E) Immunofluorescence of DUX4 in RD cells that were transfected with the indicated siRNAs and, after 24 hours, transduced with lenti-DUX4 (pRRLSIN vector backbone with a human PGK promoter driving DUX4 expression). Images were taken 42 hours following DUX4 transduction, when clear viability differences between knockdown conditions were evident. Note that siMYC appeared to have no clear effect on either nuclear localization or overall expression of DUX4 compared to the control knockdown. (F) Western blot showing DUX4 and MYC protein levels following the indicated knockdowns at 18 hours after transduction of lenti-DUX4. (G) CellTiter-Glo viability assay following the indicated knockdowns at 48 hours after transduction of lenti-DUX4. (TIF) [file pgen.1006658.s004.tif]

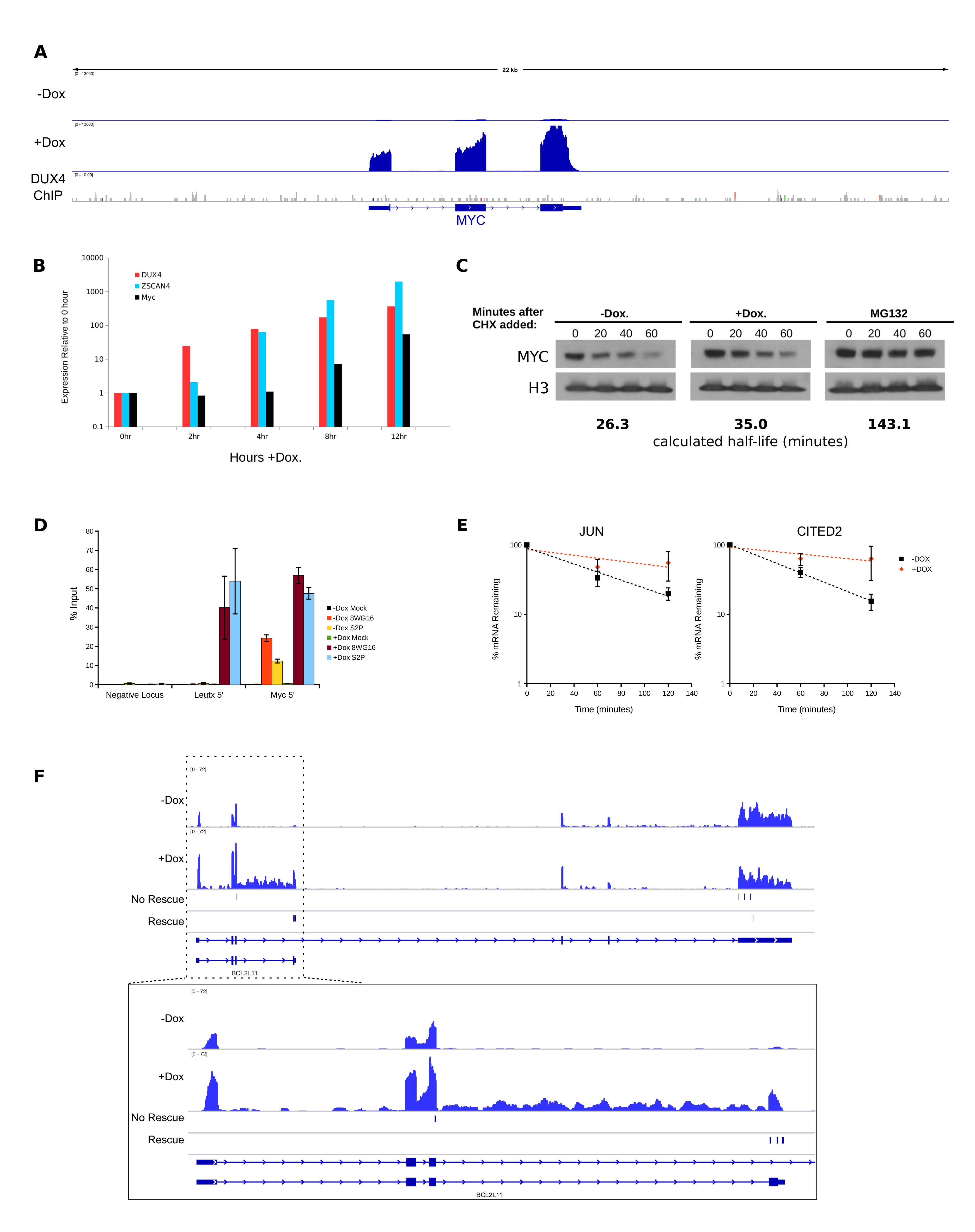

Supplement: S5 Fig — (A) Track showing RNA-seq or ChIP-seq reads mapped 22kb at and surrounding the MYC locus. Note that there is no apparent DUX4 occupancy near the canonical MYC promoter nor elsewhere. (B) RT-qPCR data of MYC and ZSCAN4 (a direct transcriptional target of DUX4) following doxycyline induction in RD-DUX4i cells. (C) Western blot for protein half-life experiment of MYC in RD-DUX4i cells. Cells were treated with or without doxycyline for 8 hours prior to the addition of the translation inhibitor, cyclohexamide (CHX). MG132, a proteosomal inhibitor, was included as a positive control and added during CHX addition. Densitometry was used to estimate relative protein levels compared to the zero-hour time point and data were fitted onto a semi-log plot in order to estimate the half-life of each condition. (D) ChIP-qPCR of unphosphorylated (8WG16) and Serine-2 phosphorylated forms of RNA polymerase II. The “negative locus” is a primer-set with no known annotated transcripts and serves as a negative control. Error bars represent the standard deviation of three replicate ChIP experiments. (E) mRNA half-life experiment as in Fig 2B. (F) RNA-seq track showing the location of the rescuing and non-rescuing BCL2L11 (BIM) pool of siRNAs in RD cells +/- doxycycline. The shorter splice form induced by DUX4 is the BIMγ isoform of BCL2L11 and the rescuing pool has three siRNA that specifically target this isoform. (TIF) [file pgen.1006658.s005.tif]

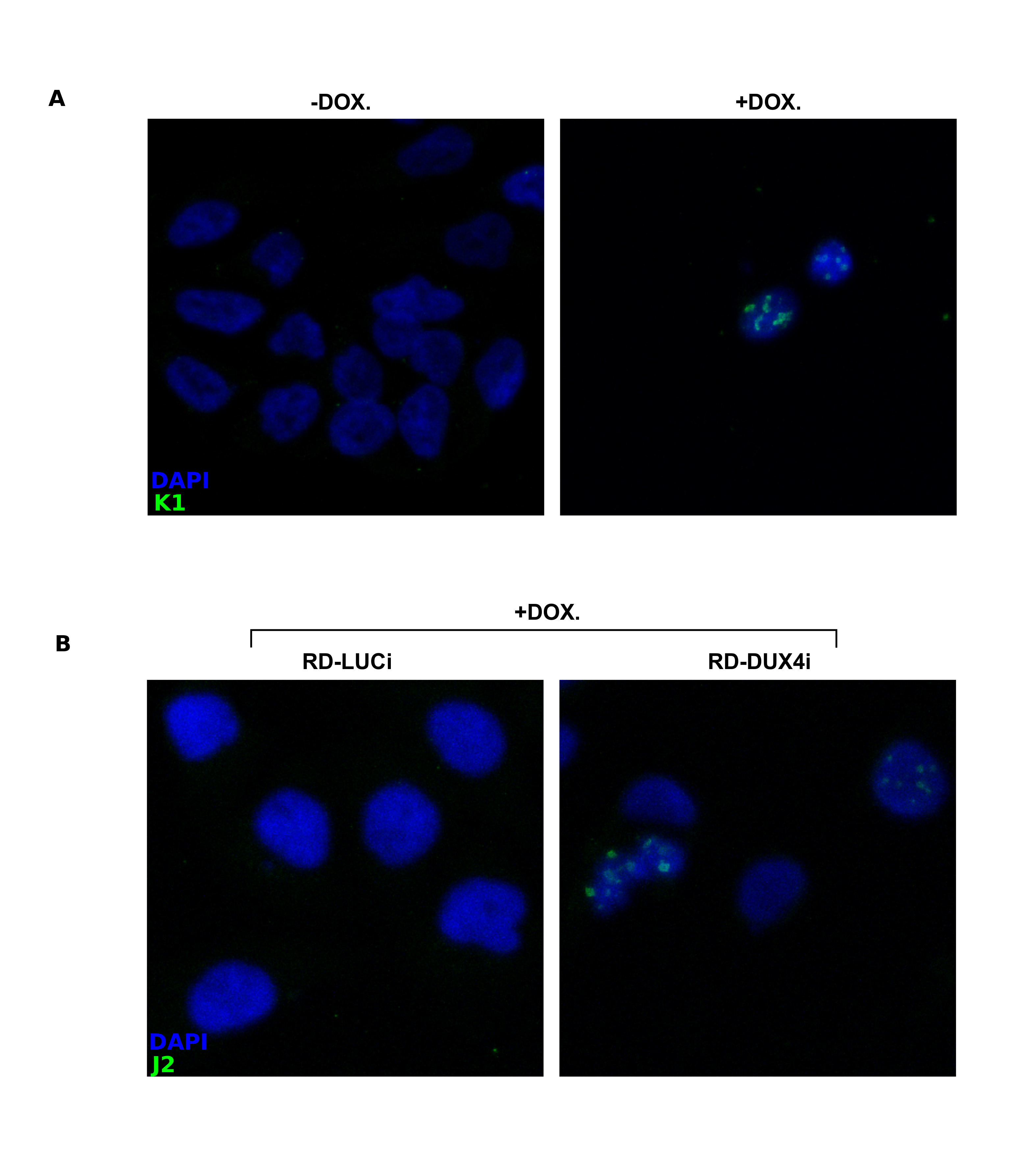

Supplement: S6 Fig — (A) Confocal microscopy immunofluorescence of RD-DUX4i cells +/- doxycyline using the K1 antibody to verify the presence of dsRNAs in DUX4 expressing cells. (B) Confocal microscopy immunofluorescence of RD-LUCi and RD-DUX4i cells with doxycycline and stained with the J2 antibody. Note that, based on previous observations, we believe induction of DUX4 occurs asynchronously in the cell population using the doxycycline-inducible system, explaining why there is not universal nuclear dsRNA positivity at the time point assayed. (TIF) [file pgen.1006658.s006.tif]

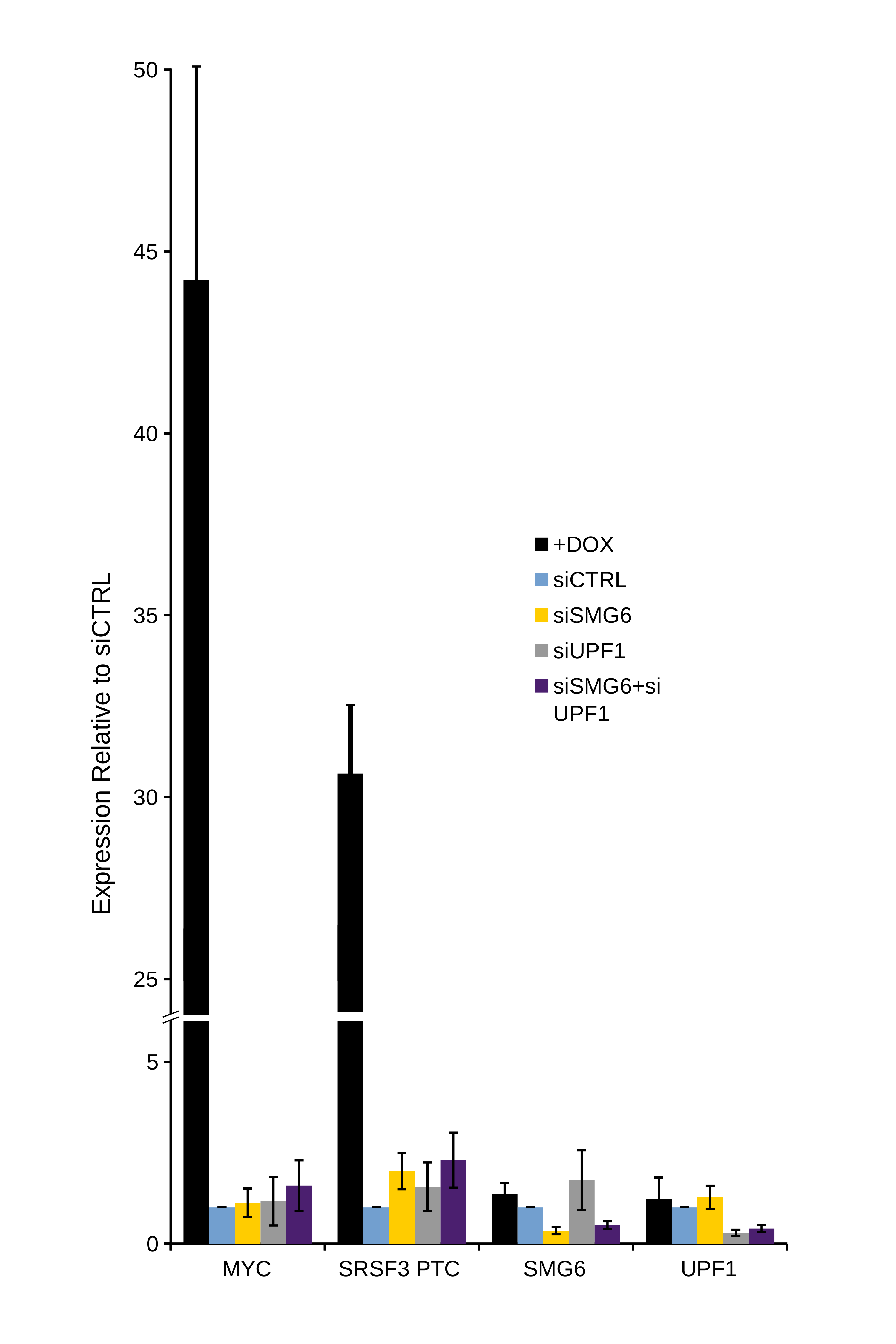

Supplement: S7 Fig — RT-qPCR data on RD-DUX4i cells either induced with doxycycline (Dox+) or uninduced in the presence of the indicated siRNA and using the primer sets annotated on the X-axis. Error bars represent the standard deviation of three separate experiments. (TIF) [file pgen.1006658.s007.tif]

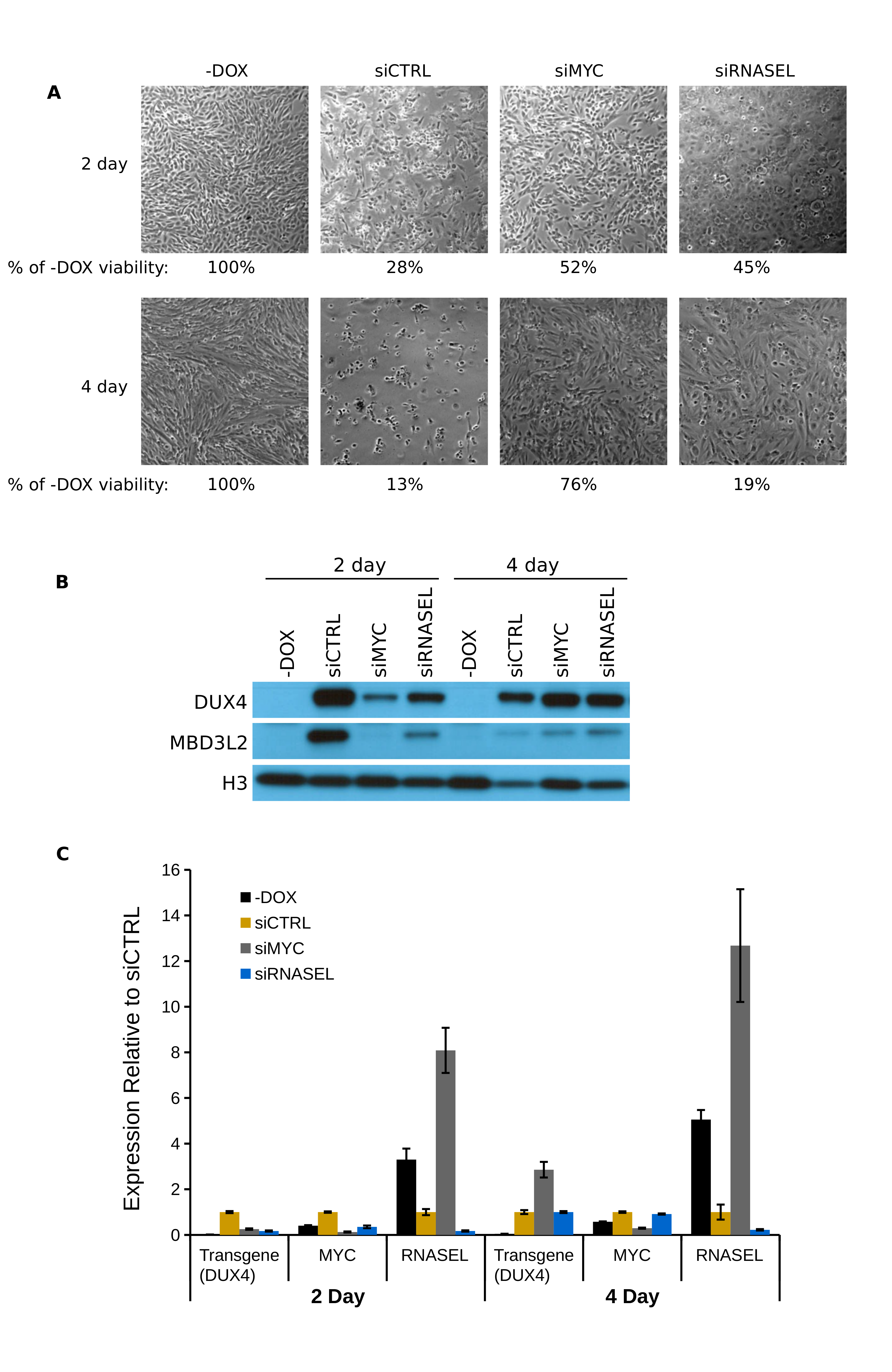

Supplement: S8 Fig — (A) Bright field images showing MB135-DUX4i cell morphology after DUX4 expression at 2 and 4 days post-induction. The ATP-based CellTiter-Glo assay gave unusually high readings in control myoblast cells for unknown reasons and thus we used viable cell counts as an alternative. (B) Western blot showing a DUX4 target gene, MBD3L2, and DUX4 transgene expression in MB135-DUX4i cells at the indicated time-points after induction. (C) RT-qPCR to confirm knockdown of target mRNAs. Data are normalized to RPL27A and shown relative to the siCTRL condition. Error bars represent the standard deviation of the mean of three replicate wells. (TIF) [file pgen.1006658.s008.tif]

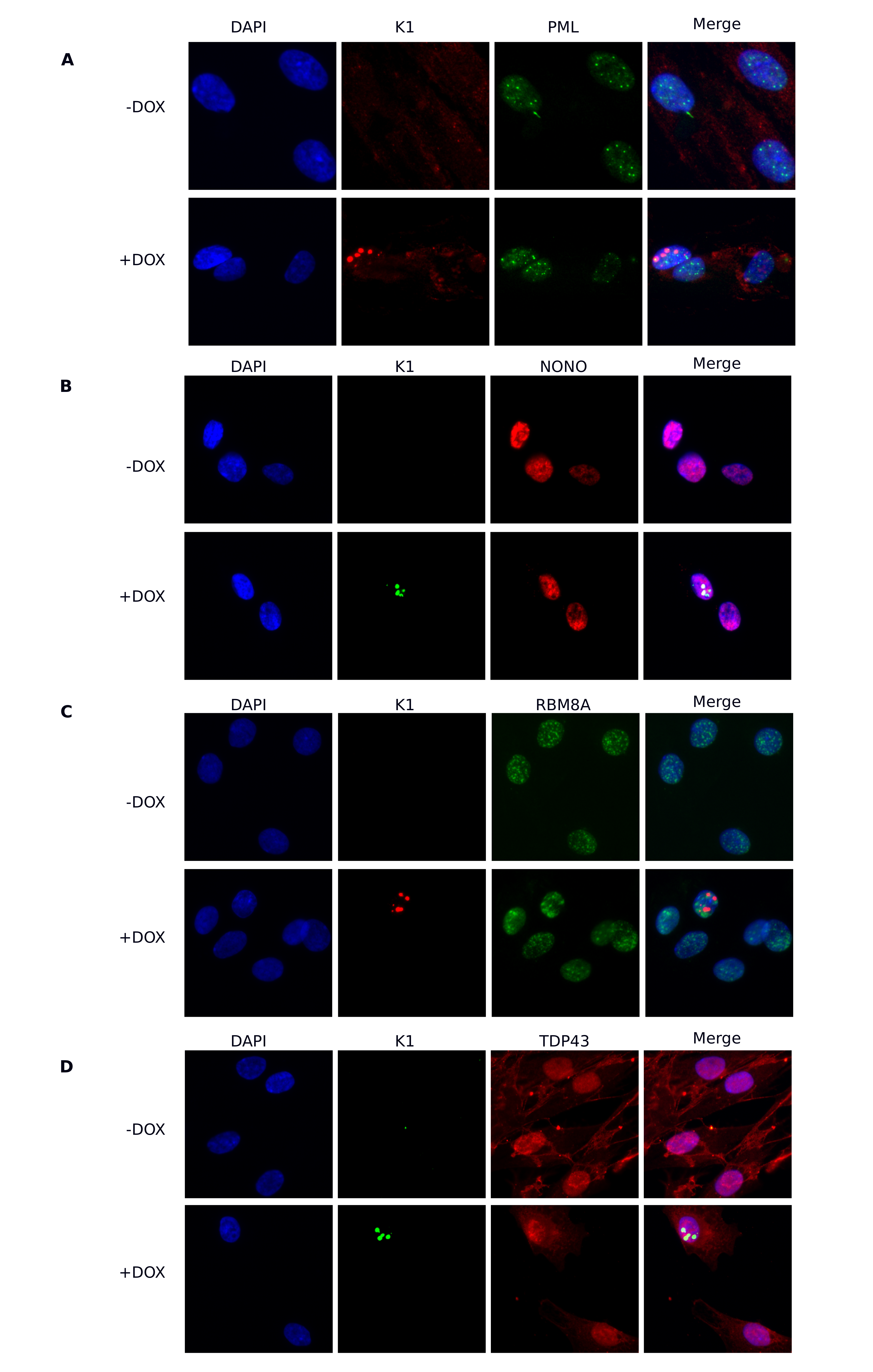

Supplement: S9 Fig — MB135-DUX4i cells were induced for 24 hours with doxycycline prior to co-staining with antibodies against dsRNA (K1) and (A) PML. (B) NONO (paraspeckles). (C) RBM8A. (D) TDP-43, none of which show exclusive overlap with dsRNA accumulation. (TIF) [file pgen.1006658.s009.tif]
